# *Pseudomonas aeruginosa* exoproducts determine antibiotic efficacy against *Staphylococcus aureus*

**DOI:** 10.1371/journal.pbio.2003981

**Published:** 2017-11-27

**Authors:** Lauren Radlinski, Sarah E. Rowe, Laurel B. Kartchner, Robert Maile, Bruce A. Cairns, Nicholas P. Vitko, Cindy J. Gode, Anne M. Lachiewicz, Matthew C. Wolfgang, Brian P. Conlon

**Affiliations:** 1 Department of Microbiology and Immunology, University of North Carolina at Chapel Hill, North Carolina, United States of America; 2 North Carolina Jaycee Burn Center, University of North Carolina at Chapel Hill, North Carolina, United States of America; 3 Marsico Lung Institute, University of North Carolina at Chapel Hill, North Carolina, United States of America; 4 Division of Infectious Diseases, University of North Carolina at Chapel Hill, North Carolina, United States of America; Universite de Lausanne Departement de Microbiologie Fondamentale, Switzerland

## Abstract

Chronic coinfections of *Staphylococcus aureus* and *Pseudomonas aeruginosa* frequently fail to respond to antibiotic treatment, leading to significant patient morbidity and mortality. Currently, the impact of interspecies interaction on *S*. *aureus* antibiotic susceptibility remains poorly understood. In this study, we utilize a panel of *P*. *aeruginosa* burn wound and cystic fibrosis (CF) lung isolates to demonstrate that *P*. *aeruginosa* alters *S*. *aureus* susceptibility to bactericidal antibiotics in a variable, strain-dependent manner and further identify 3 independent interactions responsible for antagonizing or potentiating antibiotic activity against *S*. *aureus*. We find that *P*. *aeruginosa* LasA endopeptidase potentiates lysis of *S*. *aureus* by vancomycin, rhamnolipids facilitate proton-motive force-independent tobramycin uptake, and 2-heptyl-4-hydroxyquinoline *N*-oxide (HQNO) induces multidrug tolerance in *S*. *aureus* through respiratory inhibition and reduction of cellular ATP. We find that the production of each of these factors varies between clinical isolates and corresponds to the capacity of each isolate to alter *S*. *aureus* antibiotic susceptibility. Furthermore, we demonstrate that vancomycin treatment of a *S*. *aureus* mouse burn infection is potentiated by the presence of a LasA-producing *P*. *aeruginosa* population. These findings demonstrate that antibiotic susceptibility is complex and dependent not only upon the genotype of the pathogen being targeted, but also on interactions with other microorganisms in the infection environment. Consideration of these interactions will improve the treatment of polymicrobial infections.

## Author summary

Accurate prediction of antimicrobial efficacy is essential for successful treatment of a bacterial infection. While many studies have considered the impacts of genetically encoded mechanisms of resistance, nongenetic determinants of antibiotic susceptibility during infection remain poorly understood. Here we show that a single interspecies interaction between 2 human pathogens, *S*. *aureus* and *P*. *aeruginosa*, can completely transform the antibiotic susceptibility profile of *S*. *aureus*. Through multiple distinct mechanisms, *P*. *aeruginosa* can antagonize or potentiate the efficacy of multiple classes of antibiotics against *S*. *aureus*. We identify the exoproducts responsible for altering *S*. *aureus* susceptibility to antibiotic killing, and furthermore demonstrate that these compounds are produced at varying levels in *P*. *aeruginosa* clinical isolates, with dramatic repercussions for *S*. *aureus* antibiotic susceptibility. Finally, we use a mouse model of *P*. *aeruginosa–S*. *aureus* coinfection to demonstrate that the presence of *P*. *aeruginosa* significantly alters the outcome of *S*. *aureus* antibiotic therapy in a host. These findings indicate that the efficacy of antibiotic treatment in polymicrobial infection is determined at the community level, with interspecies interaction playing an important and previously unappreciated role.

## Introduction

*S*. *aureus* is responsible for numerous chronic and relapsing infections such as osteomyelitis, endocarditis, and infections of the cystic fibrosis (CF) lung, as well as many penetrating trauma and burn infections, venous leg ulcers, pressure ulcers, and diabetic foot ulcers. These infections are notoriously difficult to treat, despite isolates frequently exhibiting full sensitivity to administered antibiotics, as measured in vitro using a Minimum Inhibitory Concentration (MIC) assay. This suggests that environmental factors present in vivo may influence the pathogen’s susceptibility to antibiotic killing. While these factors can include physical barriers to antibiotic activity, such as tissue necrosis and low vascularization at a site of infection, or bacterial replication within host phagocytes, treatment failure cannot be fully explained by poor drug penetration [1]. Instead, environmental determinants, such as interactions with the host, can induce phenotypic responses or genetic adaptations in bacteria that reduce antibiotic sensitivity [2,3].

Similarly, within complex polymicrobial communities such as those encountered in chronic skin infections, burn wound infections, and chronic colonization of the CF lung, inter- and intraspecies interactions can influence the pathogenicity and antibiotic susceptibility of individual organisms [4–6]. The presence of the fungal pathogen *Candida albicans*, for instance, can induce *S*. *aureus* biofilm formation and thus decrease the bacterium’s susceptibility to antibiotic killing [4]. Furthermore, antibiotic deactivation by resistant organisms within a population can lead to de facto resistance of all members of the community [7–10].

In such polymicrobial infections, *S*. *aureus* is commonly co-isolated with the opportunistic pathogen *P*. *aeruginosa* [11]. These co-infections are generally more virulent and/or more difficult to treat than infections caused by either pathogen alone [12–14]. The interaction between these 2 organisms is complex, with *P*. *aeruginosa* producing a number of molecules that interfere with *S*. *aureus* growth, metabolism, and cellular homeostasis. These molecules include the secondary metabolites 4-hydroxy-2-heptylquinoline-*N*-oxide (HQNO), pyocyanin, and hydrogen cyanide (HCN), all of which inhibit *S*. *aureus* respiration [15–17]. Additionally, *P*. *aeruginosa* produces rhamnolipids, biosurfactants that interfere with the *S*. *aureus* cell membrane, and an endopeptidase, LasA, that cleaves pentaglycine bridges in *S*. *aureus* peptidoglycan [18–20].

These anti-staphylococcal compounds allow *P*. *aeruginosa* to quickly eliminate *S*. *aureus* during in vitro coculture but do not prevent co-colonization in vivo. Recent findings suggest that within the CF lung, *P*. *aeruginosa* strains evolve to be less competitive with *S*. *aureus*, resulting in more stable coinfection of the same spatial niche [21]. Additionally, work by Wakeman et al. has shown that the presence of the abundant innate immune protein, calprotectin, induces a phenotypic switch in *P*. *aeruginosa* that promotes stable *P*. *aeruginosa* and *S*. *aureus* interaction through the chelation of zinc and manganese ions at the site of infection. This in turn represses *P*. *aeruginosa* metabolic toxin production, resulting in significantly less HQNO and pyocyanin [22]. Similarly, Smith et al. recently demonstrated that *S*. *aureus* can tolerate in vitro coculture with *P*. *aeruginosa* in the presence of serum albumin through the inhibition of *P*. *aeruginosa lasR* quorum sensing and thus LasA expression [23]. Despite these findings, *P*. *aeruginosa* LasA, rhamnolipids, HQNO, and pyocyanin are routinely detected at significant concentrations in burn wounds and in CF sputum samples and thus likely influence *S*. *aureus* physiology [24–28].

We hypothesized that interaction with *P*. *aeruginosa* may antagonize or potentiate *S*. *aureus* antibiotic susceptibility and could explain the frequent occurrence of treatment failure in infections involving otherwise drug-susceptible strains. Furthermore, we hypothesized that such interactions could be exploited to improve antibiotic treatment outcome. Here we demonstrate that secreted *P*. *aeruginosa* factors dramatically alter *S*. *aureus* susceptibility to killing by multiple antibiotic classes, and identify several mediators of *S*. *aureus* antibiotic antagonism or potentiation. Importantly, the production of these molecules is highly strain dependent, thus implicating the genotype of coinfecting *P*. *aeruginosa* strains as critical determinants of antibiotic treatment outcomes for *S*. *aureus* infections. Ultimately, we demonstrate in a mouse model of *S*. *aureus*, *P*. *aeruginosa* coinfection that the presence of *P*. *aeruginosa* can significantly alter the outcome of *S*. *aureus* antibiotic treatment. Overall, this work highlights the importance of considering the microbial context of the infection environment during the treatment of polymicrobial infection.

## Results

### *P*. *aeruginosa* alters *S*. *aureus* susceptibility to antibiotic killing

To investigate the impact of *P*. *aeruginosa* on *S*. *aureus* antibiotic susceptibility, we measured the bactericidal activity of 3 antibiotics against *S*. *aureus* in the presence of supernatants from 12 *P*. *aeruginosa* clinical isolates; 7 from the lungs of CF patients and 5 from burn wounds, as well as 2 laboratory strains; PAO1 and PA14. We were interested in examining how *P*. *aeruginosa*-secreted exoproducts can impact the susceptibility of *S*. *aureus* to vancomycin, tobramycin, and ciprofloxacin. Vancomycin is the frontline antibiotic for the treatment of methicillin-resistant *S*. *aureus* (MRSA). Ciprofloxacin and tobramycin are commonly used to treat *P*. *aeruginosa* during coinfection.

**Fig 1:**
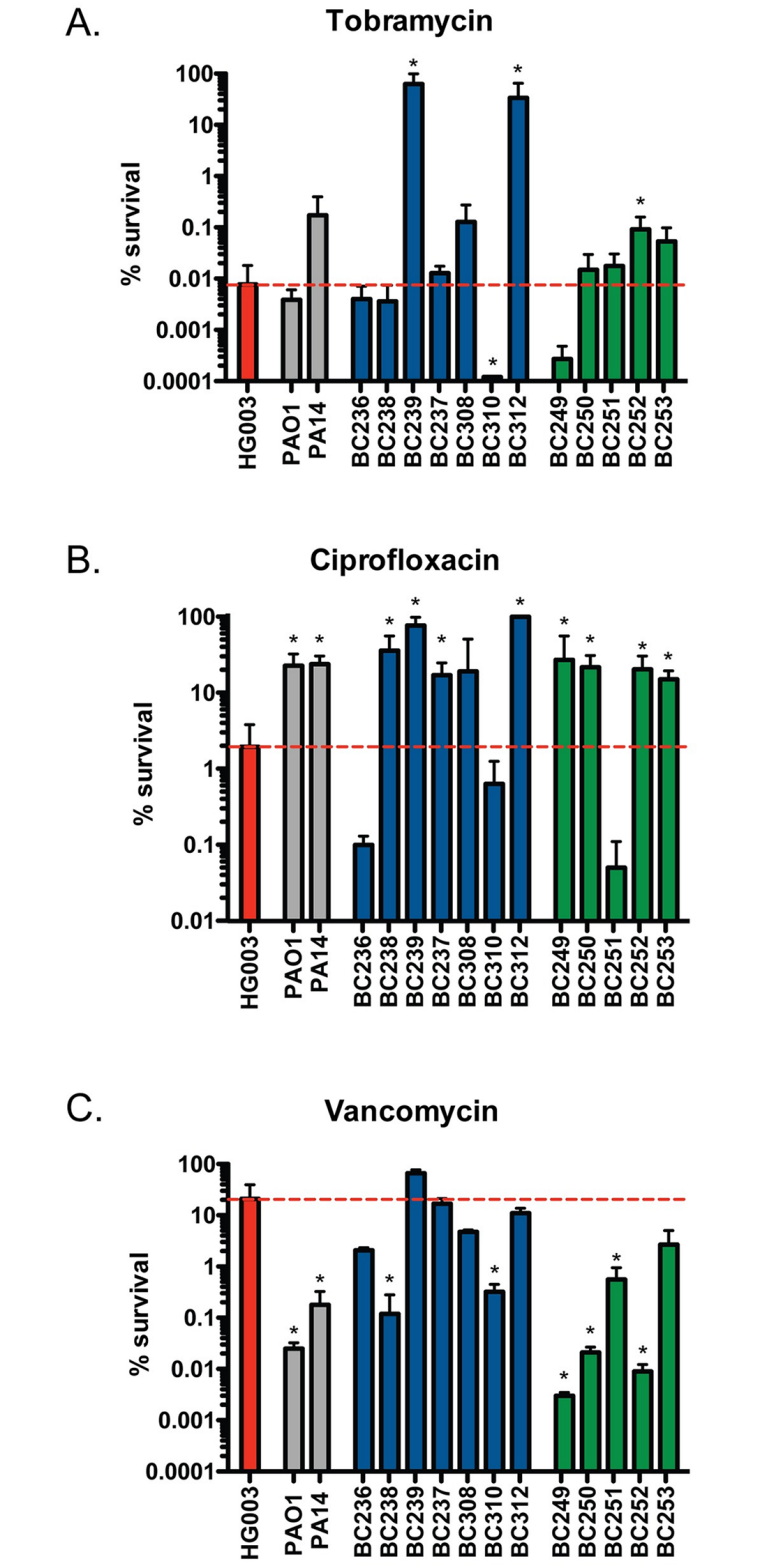
*P*. *aeruginosa* supernatant alters *S*. *aureus* antibiotic susceptibility. *S*. *aureus* strain HG003 was grown to mid-exponential phase and exposed to sterile supernatants from *S*. *aureus* HG003 (red), *P*. *aeruginosa* laboratory strains PAO1 and PA14 (grey), *P*. *aeruginosa* CF clinical isolates (blue) or *P*. *aeruginosa* burn isolates (green) for 30 min prior to addition of (A) 50 μg/ml vancomycin, (B) 58 μg/ml tobramycin or (C) 2.34 μg/ml ciprofloxacin concentrations similar to the Cmax in humans. An aliquot was removed after 24 h, washed, and plated to enumerate survivors. The dotted red line represents the number of survivors in the control culture. All experiments were performed in biological triplicate and the number of survivors following antibiotic challenge in the presence of *P*. *aeruginosa* supernatant was compared to the HG003 supernatant-treated control. Underlying data can be found in S1 Data. *p<0.05 (one-way ANOVA with Tukey’s multiple comparisons post-test analysis of surviving CFU). Error bars represent mean + sd. CF, cystic fibrosis; CFU, colony-forming units.

*S*. *aureus* strain HG003 was grown to exponential phase and treated with 500 μL of sterile supernatant from overnight (18 h) cultures of HG003 (control) or one of the 14 *P*. *aeruginosa* strains prior to antibiotic challenge. After 24 h, cells were washed and plated to enumerate survivors. We found that the individual bactericidal activities of all 3 antibiotics against *S*. *aureus* were affected by *P*. *aeruginosa* supernatants. More specifically, we observed 3 *P*. *aeruginosa* isolates that significantly protected *S*. *aureus* from killing by tobramycin (BC239, BC312, and BC252) and one *P*. *aeruginosa* isolate (BC310) induced over a 10-fold increase in tobramycin killing of *S*. *aureus* (Fig 1A). We also observed that the majority of *P*. *aeruginosa* supernatants were antagonistic towards ciprofloxacin killing (Fig 1B). Furthermore, supernatants from 8 *P*. *aeruginosa* strains (PAO1, PA14, BC238, BC310, BC249, BC250, BC251, and BC252) dramatically potentiated vancomycin killing of *S*. *aureus*, resulting in 100–1,000 times more killing than the control culture (Fig 1C). These data highlight the variable and strain-dependent influence of *P*. *aeruginosa* on the susceptibility of *S*. *aureus* to different antibiotics; however, the mechanism(s) by which *P*. *aeruginosa* alters *S*. *aureus* antibiotic susceptibility remained unclear.

### *P*. *aeruginosa* rhamnolipids increase tobramycin uptake and efficacy against *S*. *aureus*

Previous studies have shown that during coculture the presence of *P*. *aeruginosa* results in increased *S*. *aureus* resistance to tobramycin through the activity of HQNO [5]. In agreement with this, we observed that supernatants from BC239 and BC312 and BC252 protected *S*. *aureus* from tobramycin killing (Fig 1A). Paradoxically, however, we observed that the majority of our clinical isolates had no significant impact on tobramycin bactericidal activity. Even more striking, isolate BC310 appeared to potentiate tobramycin bactericidal activity against *S*. *aureus* (Fig 1A). We hypothesized that the impact of *P*. *aeruginosa* on *S*. *aureus* tobramycin susceptibility was multifactorial, with an unidentified factor increasing tobramycin bactericidal activity.

**Fig 2:**
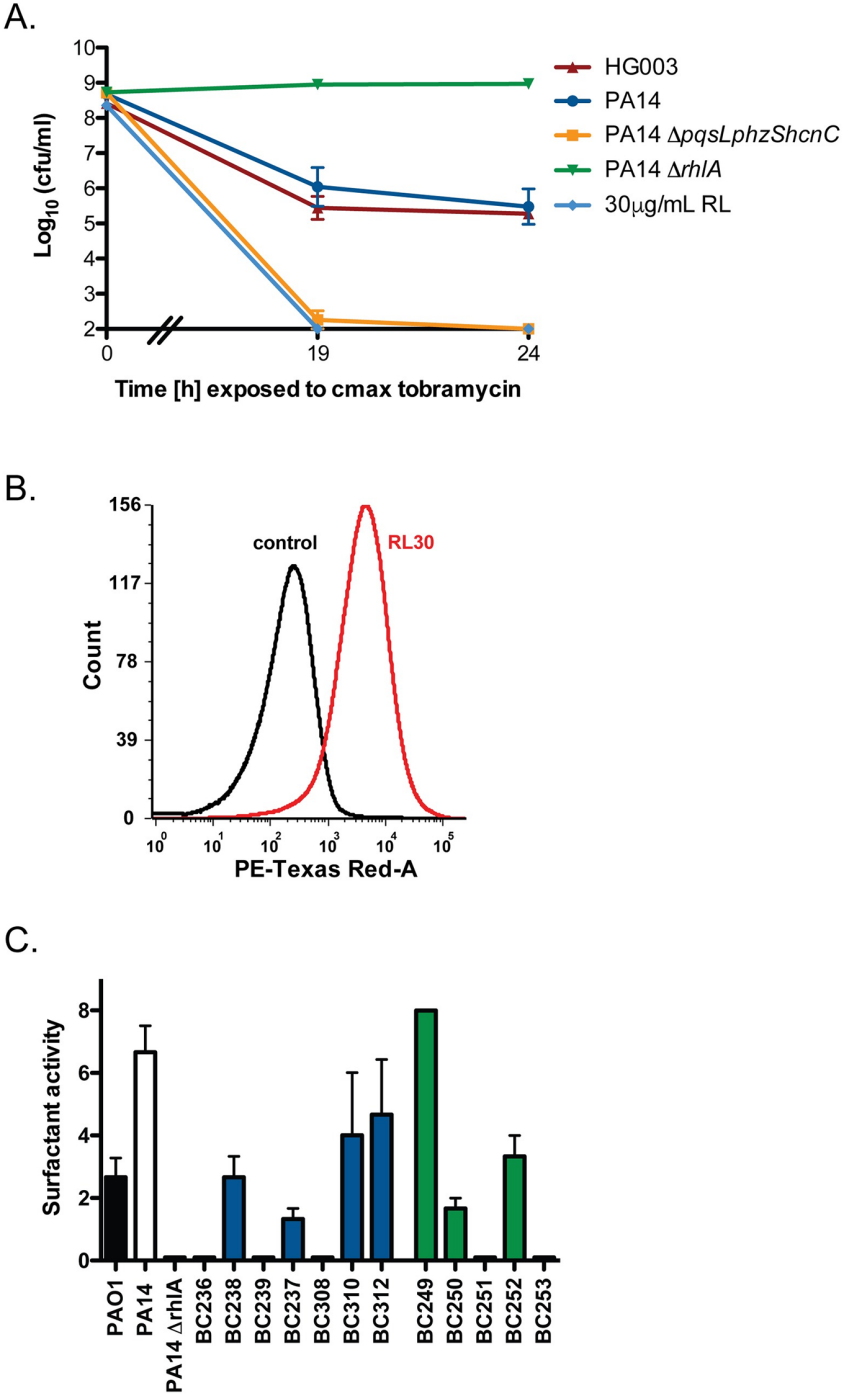
*P*. *aeruginosa* rhamnolipids potentiate aminoglycoside uptake and cell death in *S*. *aureus*. *S*. *aureus* HG003 was grown to mid-exponential phase and exposed to (A) sterile supernatants from *P*. *aeruginosa* or *S*. *aureus* or exogenous addition of rhamnolipids (30 μg/ml) before addition of tobramycin 58 μg/ml. At indicated times, an aliquot was washed and plated to enumerate survivors. (B) Texas Red-conjugated tobramycin was added to *S*. *aureus* cultures with or without 30 μg/ml rhamnolipids. Following 1 h, Texas Red-tobramycin uptake was measured by flow cytometry. (C) Rhamnolipid production present in the supernatant of *P*. *aeruginosa* PAO1, PA14, PA14 *ΔrhlA*, CF isolates (blue) or burn isolates (green) were quantified by a drop-collapse assay. Experiments were performed in biological triplicate. Underlying data can be found in S1 Data. Error bars represent mean ± sd. CF, cystic fibrosis.

Tobramycin uptake is dependent on proton-motive force (PMF) [29]. *P*. *aeruginosa* HQNO collapses *S*. *aureus* PMF by inhibiting electron transport, thus abolishing tobramycin uptake into the cell [5]. To explore the possibility that an additional factor within *P*. *aeruginosa* supernatant may influence the bactericidal activity of tobramycin against *S*. *aureus*, we examined *S*. *aureus* susceptibility to tobramycin in the presence of supernatant from a PA14 *ΔpqsLphzShcnC* strain. This strain cannot produce the respiratory toxins HQNO, pyocyanin, or HCN, all of which inhibit *S*. *aureus* respiration and deplete PMF. Strikingly, we found that PA14 *ΔpqsLphzShcnC* mutant supernatant led to the rapid eradication of a *S*. *aureus* population following tobramycin treatment (Fig 2A) (S1A Fig). Heat-inactivation of *P*. *aeruginosa* PA14 supernatant had no impact on its ability to alter tobramycin activity, ruling out heat-labile proteins as potentiators of tobramycin killing (S1B Fig).

*P*. *aeruginosa* produces surfactant molecules called rhamnolipids that inhibit growth of competing gram-positive bacteria. These amphiphilic molecules increase cell permeability by interacting with the plasma membrane [30]. We hypothesized that rhamnolipid interaction with the membrane may facilitate tobramycin entry into otherwise tolerant, PMF-depleted persister subpopulations. To investigate this possibility, we deleted the *rhlA* gene in PA14, which is essential for rhamnolipid biosynthesis. Supernatant from a PA14 *ΔrhlA* mutant conferred full protection to *S*. *aureus* against tobramycin killing (Fig 2A) (S1A Fig). Furthermore, during tobramycin treatment, the exogenous addition of a 50/50 mix of purified *P*. *aeruginosa* mono- and di-rhamnolipids at 30 μg/ml facilitated the rapid eradication of the *S*. *aureus* population and decreased the MIC of tobramycin for *S*. *aureus* 8-fold (Fig 2A) (S1 Table). This concentration is within the physiological range of rhamnolipids likely encountered by *S*. *aureus* during coinfection with *P*. *aeruginosa*, as previous work by Bjarnsholt et al. found that clinical isolates produce a range of 2.4 μg/ml to 72.8 μg/ml rhamnolipids when grown in vitro, and Read et al. reported rhamnolipid concentrations as high as 64 μg/ml in a CF lung explant [24,31]. At the concentrations used in this study, rhamnolipids did not display antibacterial activity in the absence of antibiotic (S1C Fig). Further, incubation with a similar concentration of L-rhamnose, the glycosyl head constituent of rhamnolipids, had no effect on tobramycin killing, ruling out metabolite-stimulated PMF generation as the mechanism of tobramycin potentiation (S1D Fig). Finally, we found that 30 μg/ml purified *P*. *aeruginosa* rhamnolipids led to increased uptake of Texas Red-conjugated tobramycin as determined by flow cytometry (Fig 2B).

We next measured the relative amount of HQNO and rhamnolipids produced by each *P*. *aeruginosa* isolate using mass spectrometry [32] and a drop-collapse assay, respectively [33]. We observed a large variance in the production of both HQNO (Table 1) and rhamnolipids between isolates (Fig 2C). Importantly, the potentiator of tobramycin activity, BC310, was the only strain shown to be a high rhamnolipid producer without detectable HQNO production. In contrast, strain BC239, the strongest tobramycin antagonist, was among the highest HQNO producers, and did not produce rhamnolipids. Together, these data show that *P*. *aeruginosa* has the capacity to both positively and negatively influence *S*. *aureus* tobramycin uptake and bactericidal activity through the action of rhamnolipids and respiratory toxins, respectively. The presence of these 2 opposing factors may be responsible for the apparent disconnect between the *P*. *aeruginosa*-mediated increase in tobramycin resistance reported previously [5], and lack of protection from tobramycin killing following treatment with supernatant from the majority of *P*. *aeruginosa* strains observed in this study. Indeed, deletion of either *P*. *aeruginosa* respiratory toxins or rhamnolipids in a *P*. *aeruginosa* laboratory strain resulted in supernatants that facilitate complete sterilization or protection of *S*. *aureus* cultures, respectively (Fig 2A). Furthermore, similar trends were observed when *S*. *aureus* MRSA strain JE-2 was challenged with tobramycin following treatment with *P*. *aeruginosa* supernatant, supporting the relevance of this phenomenon in the clinical treatment of *S*. *aureus* infection (S6A Fig).

**Table 1:** LC-MS/MS quantification of HQNO production in *P*. *aeruginosa* strains.

Strain	Conc. (μM)
PAO1	31.5
PA14	28.3
PA14 *ΔpqsL*	ND
BC236	ND
BC237	18.9
BC238	13.9
BC239	25.7
BC308	ND
BC310	ND
BC312	28.3
BC249	29.0
BC250	28.2
BC251	ND
BC252	47.0
BC253	9.8

**Abbreviations:** HQNO, 4-hydroxyquinoline *N*-oxide; LC-MS/MS, liquid chromatography tandem mass spectrometry. Conc, concentration; ND, not detected.

### *P*. *aeruginosa* induces multidrug tolerance in *S*. *aureus* through respiratory inhibition

In addition to the ability of HQNO to inhibit uptake of aminoglycosides, we made an interesting and somewhat unexpected observation during our investigation. HQNO production in *P*. *aeruginosa* isolates correlated perfectly with protection against ciprofloxacin killing (Fig 1B) (Table 1). As ciprofloxacin uptake is PMF-independent, we wondered if *P*. *aeruginosa* HQNO was conferring ciprofloxacin tolerance in *S*. *aureus* via an alternate mechanism.

**Fig 3:**
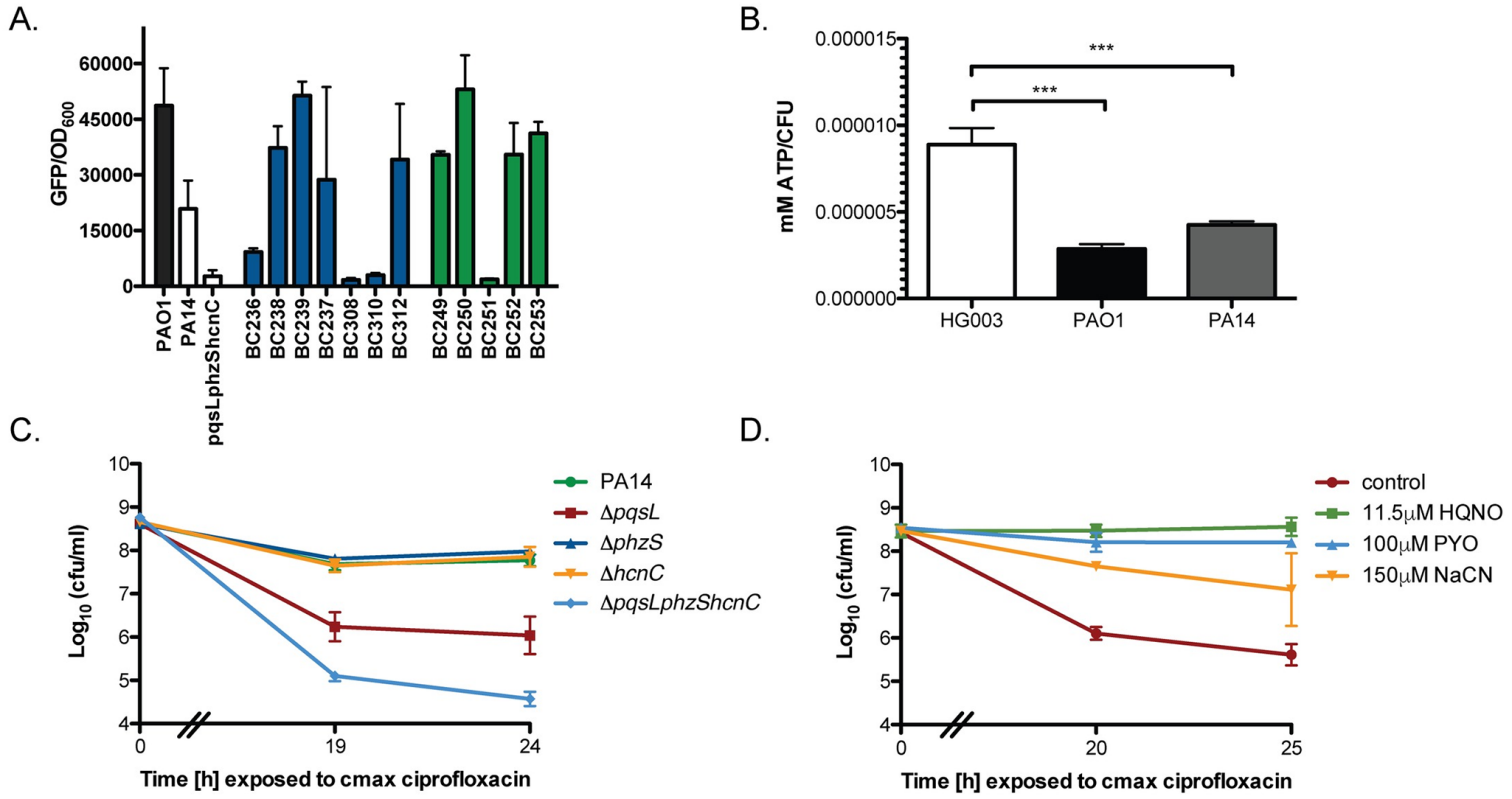
*P*. *aeruginosa* secondary metabolites inhibit *S*. *aureus* aerobic respiration resulting in a drop in intracellular ATP and protection from ciprofloxacin killing. (A) *S*. *aureus* strain HG003 harboring plasmid P*pflB*∷*gfp* was grown to mid-exponential phase and treated with supernatant from *P*. *aeruginosa* PAO1, PA14, CF isolates (blue) or burn isolates (green), for 30 min. OD_600_ and *gfp* expression levels were determined after 16 h using a Biotek Synergy H1 microplate reader. (B) Intracellular ATP was measured after 1.5 h incubation with supernatant. ***p < 0.0005 (one-way ANOVA with Tukey’s multiple comparison post-test). (C) *S*. *aureus* strain HG003 was grown to mid-exponential phase in MHB media and pre-treated with sterile supernatants from *P*. *aeruginosa* strains PA14 wild-type or its isogenic mutants or (D) physiologically-relevant concentrations of HQNO, PYO, or NaCN for 30 min prior to antibiotic challenge [26,27]. At indicated times, an aliquot was washed and plated to enumerate survivors. All experiments were performed in biological triplicate. Underlying data can be found in S1 Data. Error bars represent mean ± sd. CF, cystic fibrosis; CFU, colony-forming units; GFP, green fluorescent protein; HQNO, 4-hydroxyquinoline *N*-oxide; MHB, Mueller-Hinton broth; NaCN, sodium cyanide; OD, optical density; PYO, pyocyanin.

Antibiotic tolerance generally refers to a population-wide decrease in antibiotic susceptibility, often following exposure to external mediators of bacterial metabolism or physiology. In contrast, persister cells are generally described as antibiotic-tolerant subpopulations that form stochastically in an otherwise susceptible population. We recently demonstrated that both phenomena are specifically associated with cells entering a low ATP state [34,35]. Subpopulations of low energy cells give rise to persisters, while changes in the environment can lead to a low energy antibiotic-tolerant state in the entire population. HQNO inhibits respiration, the most efficient mechanism for ATP generation in *S*. *aureus*. We hypothesized that *P*. *aeruginosa* inhibition of *S*. *aureus* respiration induces a low ATP, multidrug-tolerant state of the entire population. In support of this, no protection from antibiotic killing was observed following pre-treatment with PA14 supernatant during anoxic growth (S2A Fig). We then cloned the fermentation-specific promoter for pyruvate acetyltransferase *(pflB)* from *S*. *aureus* upstream of *gfp* in a low-copy plasmid. Expression of *pflB* only occurs under anaerobic conditions or when respiration is inhibited [36]. We found that transcription of the *pflB* promoter was activated in response to supernatant from all of the *P*. *aeruginosa* strains with the exception of PA14 *ΔpqsLphzShcnC* (negative control) and 4 of the clinical isolates, BC236, BC308, BC310, and BC251. Importantly, these were the only clinical isolates that did not induce significant protection from ciprofloxacin killing (Fig 1B). Activation of *pflB* during aerobic growth demonstrates that respiration is inhibited in these conditions (Fig 3A). Direct intracellular ATP quantification of cultures treated with *P*. *aeruginosa* or *S*. *aureus* supernatant revealed that *P*. *aeruginosa* supernatant induces significant depletion of *S*. *aureus* intracellular ATP (Fig 3B).

We found that mutation of *pqsL* (HQNO negative) drastically reduced the capacity of PA14 supernatant to protect *S*. *aureus* from ciprofloxacin killing, suggesting tolerance to ciprofloxacin killing is mediated by HQNO (Fig 3C). Individually, mutations to the biosynthetic pathways for pyocyanin (*phzS*) and hydrogen cyanide (*hcnC)* had no influence on *P*. *aeruginosa*-conferred protection from ciprofloxacin killing. However, supernatants from a *ΔpqsLphzS* and a respiratory toxin-null mutant (*ΔpqsLphzShcnC)* were further reduced in their capacity to protect *S*. *aureus* from ciprofloxacin killing (S2B Fig) (Fig 3C). Together, these data demonstrate that *P*. *aeruginosa* confers protection from ciprofloxacin killing to *S*. *aureus* through respiration inhibition and depletion of ATP. Further, treatment with HQNO, pyocyanin, and HCN at concentrations detected within the sputum of CF patients with active *P*. *aeruginosa* infection [26,27,37] induced tolerance of *S*. *aureus* to ciprofloxacin (Fig 3D). Surprisingly, similar levels of tolerance were observed for other classes of antibiotics including tobramycin and vancomycin, with HQNO inducing the most robust tolerance to antibiotic killing (S2C and S2D Fig).

### *P*. *aeruginosa* LasA endopeptidase potentiates vancomycin bactericidal activity against *S*. *aureus*

**Fig 4:**
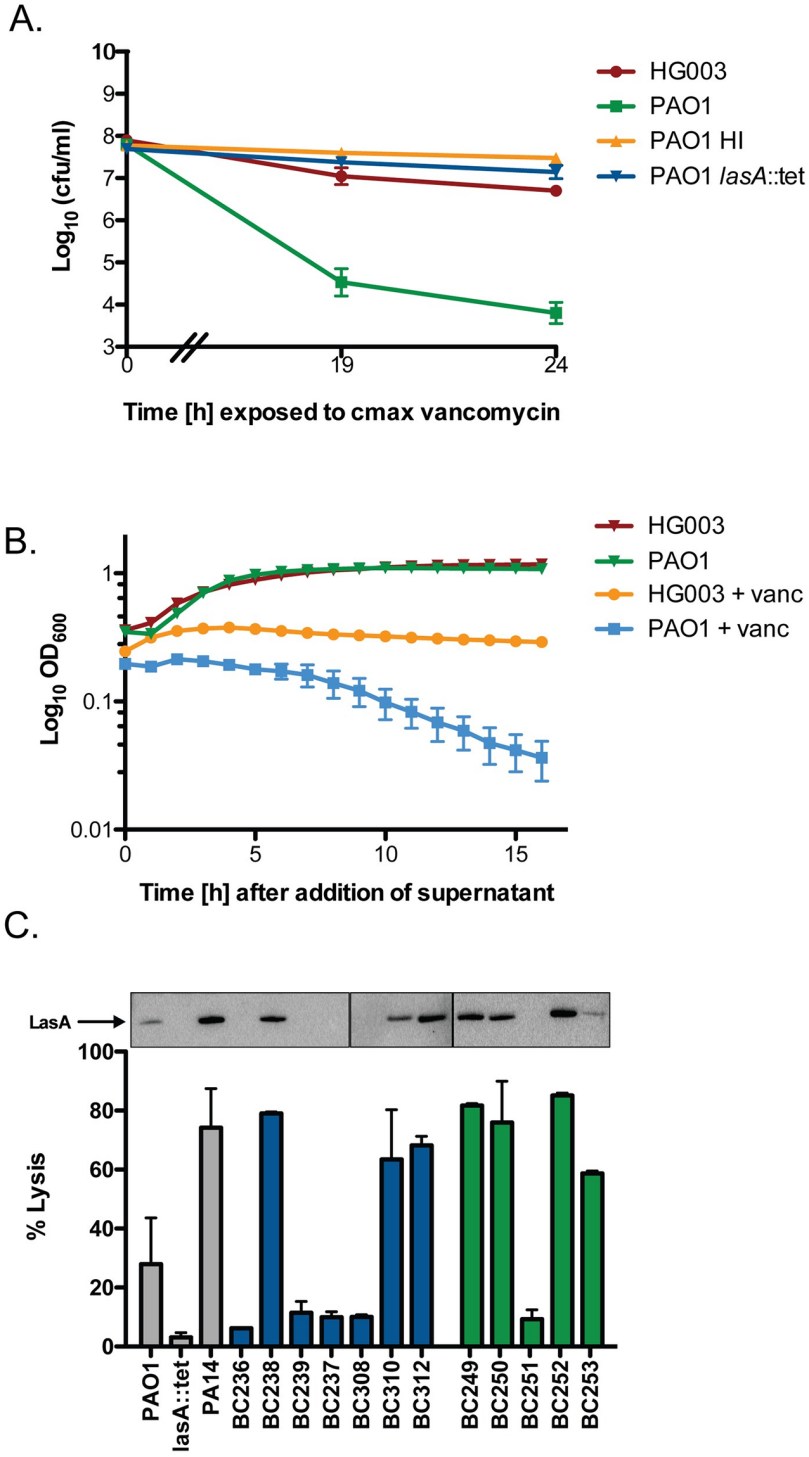
*P*. *aeruginosa* supernatant potentiates killing by vancomycin via the LasA endopeptidase. *S*. *aureus* HG003 was grown to mid-exponential phase and exposed to sterile supernatants for 30 min prior to addition of vancomycin 50 μg/ml. Where indicated, PAO1 supernatant was heat inactivated at 95°C for 10 min. (A) At indicated times, an aliquot was removed, washed, and plated to enumerate survivors or (B) 100 μl cells were added to a 96-well plate and lysis was measured at OD_600_ every hour for 16 h. (C) LasA present in the supernatant of *P*. *aeruginosa* PAO1, PA14, CF isolates (blue) or burn isolates (green) was quantified by western blot and the ability of each supernatant to lyse heat-killed *S*. *aureus* HG003 cells after 2 h. All experiments were performed in biological triplicate. Underlying data can be found in S1 Data. Error bars represent mean ± sd. CF, cystic fibrosis.

The presence of purified HQNO protects *S*. *aureus* from vancomycin killing (S2D Fig). However, *P*. *aeruginosa* supernatant from the majority of isolates tested significantly potentiated vancomycin killing of *S*. *aureus* (Fig 1C). We hypothesized that, similar to what was observed with *S*. *aureus* susceptibility to tobramycin, an additional factor present in *P*. *aeruginosa* supernatant is capable of overcoming the protective effects of HQNO to potentiate vancomycin killing of *S*. *aureus*. Heat denaturation of PAO1 supernatant completely abrogated the potentiating effect, suggesting the involvement of heat-labile extracellular protein(s) in the phenotype (Fig 4A). Bacteriolytic assays revealed that the PAO1 supernatant combined with vancomycin-induced dramatic lysis of the population that was absent in the presence of either factor alone (Fig 4B). This led us to examine the potential role of the *P*. *aeruginosa* extracellular lytic enzyme, LasA, in mediating vancomycin killing. LasA cleaves pentaglycine cross bridges in *S*. *aureus* peptidoglycan and has been shown to attack the cell wall of *S*. *aureus* during in vivo competition [18]. We examined the capacity of supernatant from a PAO1 *lasA* mutant to potentiate vancomycin killing. The *lasA* mutant supernatant did not potentiate killing by vancomycin compared to a 3-log reduction in *S*. *aureus* cfu in the presence of the PAO1 wild-type supernatant (Fig 4A) (S4A Fig). Similar trends were observed in a *S*. *aureus* MRSA strain JE-2 (S6B Fig). As it was previously shown that *S*. *aureus* can degrade HQNO [38], the absence of which could result in a more dramatic LasA-dependent potentiation effect in our supernatant experiments, we examined vancomycin killing in a coculture model where *P*. *aeruginosa* is present to continually produce HQNO. Again, we found that the presence of wild-type PAO1 resulted in a 3-Log reduction in cfu following vancomycin challenge, and that this potentiation was not observed in the presence of a PAO1 *lasA* mutant, where we observed 100-fold more survivors at 24 h (S7 Fig).

Next, we measured the levels of LasA in the supernatants of each clinical isolate via western blot and an additional assay developed previously to quantify LasA activity [39] (Fig 4C). Seven of the clinical isolates and both laboratory strains were positive for LasA. Of these, only BC253, the lowest LasA producer, and BC312, a high HQNO producer, did not induce at least a 10-fold increase in killing by vancomycin (Fig 1C). Of the 5 LasA negative strains, only one, BC251, significantly potentiated vancomycin killing, although no lysis of the culture was observed (S4B Fig). These data suggest that *P*. *aeruginosa* potentiates the vancomycin killing of *S*. *aureus* via at least 2 distinct mechanisms, only one of which is LasA-dependent.

### *P*. *aeruginosa* potentiates vancomycin killing in a mouse model of *P*. *aeruginosa/S*. *aureus* coinfection

Our observation that purified HQNO induces multidrug tolerance in *S*. *aureus* agrees with recent findings that *P*. *aeruginosa* protects *S*. *aureus* biofilm from vancomycin killing [40]. However, we have demonstrated that under planktonic growth conditions the protective effects of *P*. *aeruginosa* HQNO on *S*. *aureus* vancomycin susceptibility (S2D Fig) can be overcome by the lytic activity of LasA to potentiate vancomycin killing. In order to determine whether the protective effects of HQNO or the potentiating effects of LasA predominated in vivo, we adapted a previously described murine model of burn injury for *S*. *aureus* and *P*. *aeruginosa* coinfection [41]. Briefly, groups of mice were inflicted with a 20% total body surface area burn, then after 24 h were infected subcutaneously at the wound site with approximately 10^5^ CFU *S*. *aureus*, HG003 alone, or in combination with 10^3^ PAO1 or 10^3^ PAO1 *lasA*∷tet. Mice were then treated daily with vancomycin and harvested 72h post infection.

**Fig 5:**
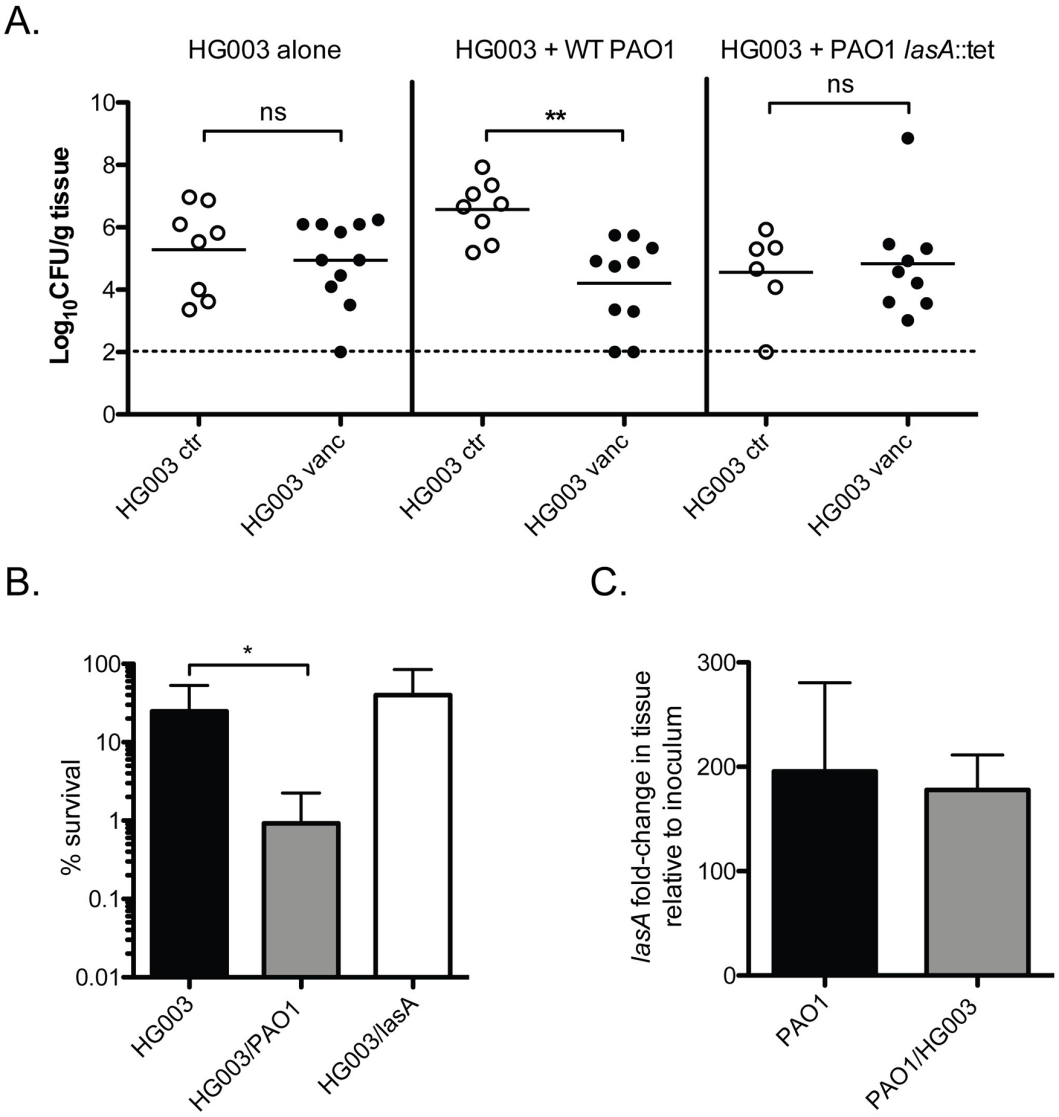
*P*. *aeruginosa* potentiates vancomycin killing of *S*. *aureus* in a murine model of coinfection. Approximately 1 x 10^5^ CFU *S*. *aureus* strain HG003 was administered subcutaneously alone or in combination with approximately 1 x 10^3^ CFU *P*. *aeruginosa* PAO1 or PAO1 *lasA*∷*tn* 24 h after burn. Mice were left untreated or administered 110 mg/kg vancomycin subcutaneously once daily for 2 d. Mice were sacrificed 48 h post infection. (A) Tissue biopsies at the site of infection were harvested and homogenized and *S*. *aureus* burdens were enumerated on selective media. Data for each group are compiled from 2 independent experiments. (*n* = 6–10 mice per group) *p < 0.05, ***p < 0.005 (Mann-Whitney test). (B) Relative percentage survival for HG003 in each condition was calculated by dividing the CFU/g tissue of mice treated with vancomycin by the average CFU/g tissue of untreated mice. Maximum percentage survival is 100%. Data for each group are compiled from 2 independent experiments. (C) Expression of *lasA* in tissue from mono- (PAO1 alone) and coinfected (PAO1/HG003) mice relative to the starting inoculum measured by qRT-PCR. Underlying data can be found in S1 Data. *p < 0.05 (Student *t* test). Error bars represent mean + sd. CFU, colony-forming units; ctr, control; qRT-PCR, quantitative reverse transcription PCR; vanc, vancomycin; WT, wild-type.

LasA has been shown to mediate *P*. *aeruginosa* epithelial cell invasion and has been shown to be essential for corneal infections [42,43]. Interestingly, in our burn model, it appeared that the presence of PAO1 resulted in a higher burden of *S*. *aureus*, which is also dependent on *lasA*. However, for this study, we were interested solely on the impact of PAO1 presence on vancomycin sensitivity of *S*. *aureus*. While we observed no significant vancomycin efficacy in *S*. *aureus* mono-infected mice, relative to an untreated control group, we observed a 2-Log reduction in *S*. *aureus* burden following vancomycin treatment in mice coinfected with *P*. *aeruginosa* PAO1 (Fig 5A and 5B). Furthermore, no potentiation of vancomycin killing was observed in mice coinfected with the PAO1 *lasA* transposon mutant (Fig 5A and 5B). Importantly, *P*. *aeruginosa* appeared to be unaffected by vancomycin treatment, and burden was similar for both wild type and PAO1 *lasA*∷*tet*-infected mice (S8 Fig). Finally, we observed that PAO1 transcription of *lasA* is strongly up-regulated (approximately 200-fold) during in vivo coinfection (Fig 5C). Up-regulation of *lasA* transcription was also observed during *P*. *aeruginosa* monoinfection, suggesting that *lasA* expression is independent of the presence or absence of *S*. *aureus* during burn wound infection.

Together, these data demonstrate that the presence of *P*. *aeruginosa* can potentiate vancomycin killing of *S*. *aureus* during infection through the production of LasA. To our knowledge, these data represent the first evidence of *P*. *aeruginosa* altering *S*. *aureus* antibiotic susceptibility in vivo and underlines the importance of deciphering interspecies interactions to improve the antibiotic treatment of polymicrobial infections.

## Discussion

**Fig 6:**
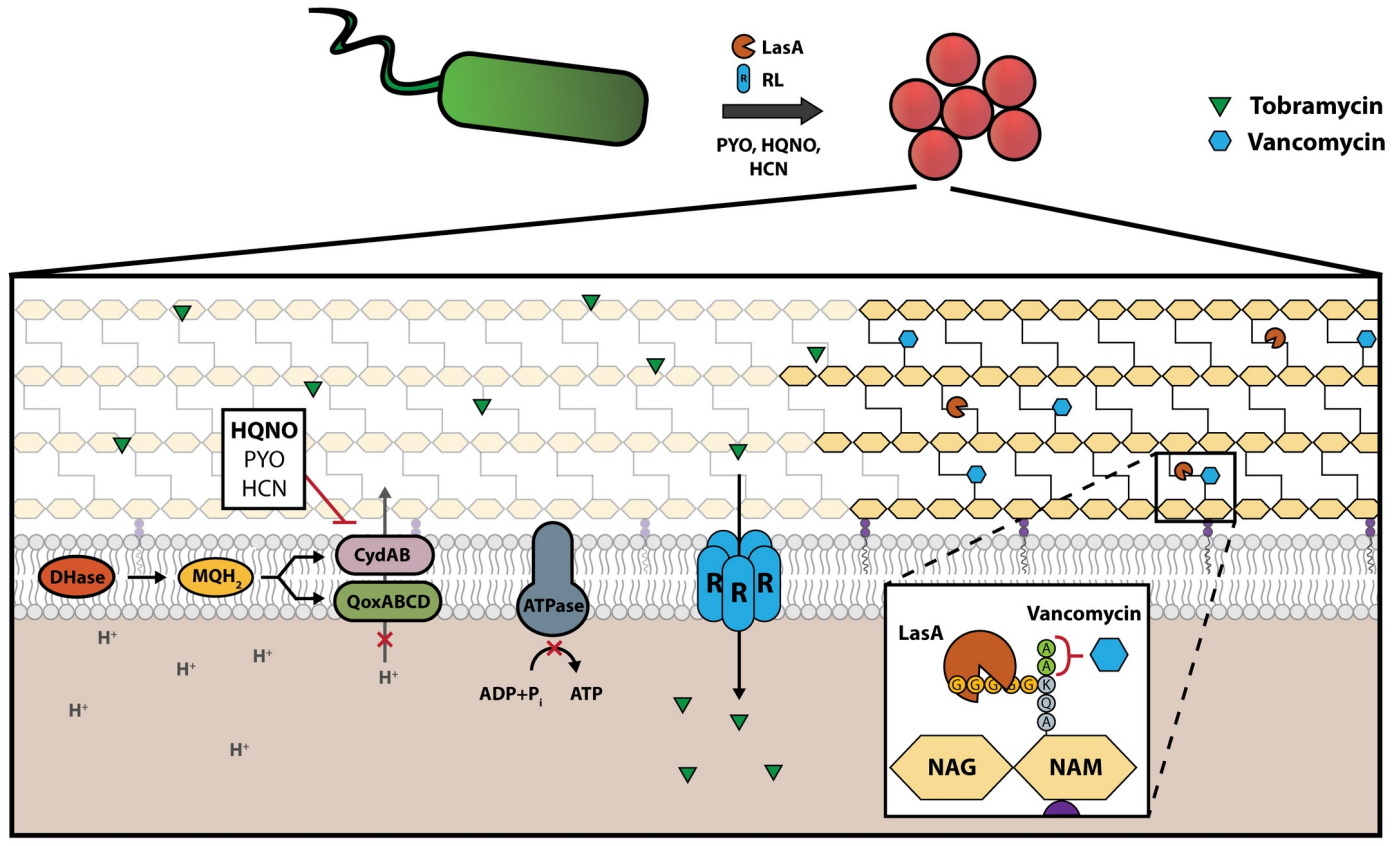
*P*. *aeruginosa*-mediated alteration of *S*. *aureus* antibiotic susceptibility. *P*. *aeruginosa* exoproducts PYO, HQNO, and HCN inhibit *S*. *aureus* electron transport, leading to collapse of PMF and inhibition of the F_1_F_0_ ATPase leading to a decrease in *S*. *aureus* antibiotic susceptibility. Conversely, *P*. *aeruginosa* RLs intercalate into the plasma membrane-forming pores that permit aminoglycoside entry into the cell in a PMF-independent manner, while *P*. *aeruginosa* endopeptidase LasA cleaves pentaglycine crosslinks between peptidoglycan molecules of the cell wall, increasing vancomycin-mediated lysis of *S*. *aureus*. HCN, hydrogen cyanide; HQNO, 2-heptyl-4-hydroxyquinoline *N*-oxide; NAG, *N*-acetylglucosamine; NAM, *N*-acetylmuramic acid; PMF, proton-motive force; PYO, pyocyanin; RL, rhamnolipids.

Polymicrobial infections are associated with exacerbated morbidity, accelerated disease progression and poor treatment outcome [44–47]. Antibiotic therapies are often selected to specifically target individual pathogens within a polymicrobial community without consideration of how interspecies interactions may alter a target organism’s antibiotic susceptibility. *S*. *aureus* and *P*. *aeruginosa* are 2 major human pathogens that frequently coexist within chronically colonized patients, and these infections are often impossible to resolve through conventional antibiotic therapy. We find that *P*. *aeruginosa* dramatically alters the susceptibility of *S*. *aureus* to the killing activities of commonly used and clinically relevant antibiotics through 3 distinct pathways governed by rhamnolipids, HQNO, and LasA, and that these molecules are produced at different levels by *P*. *aeruginosa* clinical isolates resulting in vastly different impacts on antibiotic efficacy against *S*. *aureus* (Fig 6; summarized in S2 Table). We found that *P*. *aeruginosa* staphylolytic activity correlates with vancomycin potentiation, and that *P*. *aeruginosa* HQNO production correlates with ciprofloxacin antagonism (S9A and S9B Fig). Correlation analysis with rhamnolipid production is not appropriate as the measurement of biosurfactant activity is qualitative. Overall, our results imply that antibiotic efficacy is strongly influenced by interactions between bacterial species, which may have major implications for future susceptibility determination and antibiotic treatment of polymicrobial infection.

Aminoglycosides are used routinely for the treatment of *P*. *aeruginosa* infection. Though aminoglycosides are effective against susceptible populations of *S*. *aureus*, bactericidal activity is limited against anaerobic, small colony variant (SCV), biofilm-associated, or persister subpopulations due in part to decreased respiration and thus PMF-dependent drug uptake [48,49]. Stimulating tobramycin uptake has been proposed as a way to eradicate these recalcitrant populations [49]. We have observed that *P*. *aeruginosa*-produced rhamnolipids sensitize the entire *S*. *aureus* population to tobramycin killing, leading to total eradication of otherwise tolerant populations. *P*. *aeruginosa* rhamnolipids may represent a promising new avenue for potentiating aminoglycoside killing of recalcitrant *S*. *aureus* and possibly other bacterial populations. Interestingly, a recent study has revealed that *S*. *aureus* increases tobramycin resistance in *P*. *aeruginosa* in an in vitro biofilm model, further emphasizing the importance of interaction between these organisms in dictating aminoglycoside susceptibility [50].

Vancomycin is a frontline antibiotic in the treatment of MRSA. To exert bactericidal activity against *S*. *aureus*, vancomycin must specifically bind the D-Ala-D-Ala residues of lipid II during cell wall biosynthesis [51]. However, vancomycin will also bind D-Ala-D-Ala residues of mature peptidoglycan. Thus, vancomycin exhibits limited bactericidal activity against dense populations of *S*. *aureus* cells due to the increased number of “decoy” targets available in late exponential or stationary phase populations of cells. Our data demonstrate that through LasA, *P*. *aeruginosa* can restore vancomycin efficacy against otherwise tolerant *S*. *aureus* populations. In support of this, we found that strains capable of increasing vancomycin lysis of *S*. *aureus* were LasA producers while the inert strains, generally, were not. This variance in LasA production may be due to mutations in *lasR*, an activator of *lasA* expression, which acquires mutations at high frequency during chronic *P*. *aeruginosa* infection [52]. We hypothesize that the combined action of cell wall degradation by LasA and inhibition of de novo peptidoglycan biosynthesis by vancomycin leads to cell wall destruction and a potent bactericidal effect.

*P*. *aeruginosa* HQNO induces tolerance of *S*. *aureus* to multiple antibiotic classes through respiration inhibition and depletion of intracellular ATP. Recent work by Orazi et al. found that in a bronchial epithelial tissue culture system, *P*. *aeruginosa* inhibited the killing activity of vancomycin through HQNO [40]. In agreement with these findings, we observed that the addition of exogenous HQNO protects *S*. *aureus* from vancomycin killing (S2D Fig) However, during planktonic growth the protective effect of HQNO was overshadowed by LasA-mediated potentiation of vancomycin killing. We were interested in examining whether *P*. *aeruginosa* antagonized or potentiated vancomycin in vivo, and found that *P*. *aeruginosa* expresses LasA at high levels during infection, and significantly potentiates the activity of vancomycin against *S*. *aureus*. This effect was abrogated in a *P*. *aeruginosa lasA* mutant suggesting that LasA plays a role in potentiating vancomycin killing of *S*. *aureus* during polymicrobial infection.

Similarly, we observed in vitro that *P*. *aeruginosa*-produced rhamnolipids can negate the protective effect of HQNO to restore or even increase *S*. *aureus* susceptibility to tobramycin killing. The opposing influences of HQNO and rhamnolipids on aminoglycoside activity against *S*. *aureus* may have major implications for aminoglycoside treatment of *S*. *aureus* during coinfection where production of one factor may dominate, leading to inhibition or potentiation of tobramycin activity against *S*. *aureus*. Indeed, we observed that clinical isolates produce a range of HQNO, LasA, and rhamnolipids, and production of each factor determines an isolate’s ability to potentiate or antagonize antibiotic killing.

*P*. *aeruginosa* strain variation and the impact of this variation on *S*. *aureus* antibiotic susceptibility suggests that a personalized approach to antibiotic therapy may be necessary to identify the ideal therapy to eradicate infection in an individual patient based on the genotype of *S*. *aureus* and the genotype of the bacteria it’s interacting with. Recent work has demonstrated that within the infectious environment, the production of HQNO, LasA, and rhamnolipids is highly variable. *P*. *aeruginosa* isolates from chronic CF infections frequently harbor mutations associated with decreased quorum-sensing activities and increased alginate production [52]. These mutations are attributed to the conversion to a mucoidal phenotype of *P*. *aeruginosa* that is significantly less competitive with *S*. *aureus* [53]. *P*. *aeruginosa* mucoidy is rarely associated with acute infection, thus the impact of *P*. *aeruginosa* on antibiotic susceptibility of *S*. *aureus* may differ during acute versus chronic coinfection. Future studies are necessary to identify genetic hallmarks of *P*. *aeruginosa* strains that potentiate or antagonize the activities of different antibiotic classes against *S*. *aureus* towards the goal of improving antibiotic efficacy against currently unresolvable coinfections.

It has long been observed that *S*. *aureus* is the dominant pathogen in the early life of CF patients with *P*. *aeruginosa* eventually dominating later in life [54]. It is interesting to consider a possible role of altered antibiotic susceptibility of *S*. *aureus* contributing to these dynamics, where vancomycin or tobramycin treatment in the presence of LasA- or rhamnolipid-producing *P*. *aeruginosa* strains may be particularly efficacious, resulting in a decrease in relative *S*. *aureus* abundance.

In summary, we characterized 3 distinct *P*. *aeruginosa*-mediated pathways altering *S*. *aureus* antibiotic susceptibility. HQNO induces a low-energy multidrug-tolerant state while LasA and rhamnolipids overcome this tolerance in cooperation with vancomycin and tobramycin, respectively. Exploitation of these newly discovered pathways may lead to better prediction of antibiotic efficacy in vivo and improved treatments for chronic *S*. *aureus* infection.

## Materials and methods

### Ethics statement

*P*. *aeruginosa* CF isolates were provided by an IRB-approved biospecimen bank (IRB#02–0948). *P*. *aeruginosa* burn wound isolates were from a previous study and use was deemed exempt by IRB study number #17–0836. All mice used in the study were maintained under specific pathogen-free conditions in the Animal Association of Laboratory Animal Care-accredited University of North Carolina Department of Laboratory Animal Medicine Facilities. All protocols were approved by the Institutional Animal Care and Use Committee at the University of North Carolina, protocol number 17–141, and all experiments were performed in accordance with the National Institutes of Health. Animals were anesthetized by inhalation of vaporized isoflurane. A subcutaneous injection of morphine was given prior to burn injury for pain control, and an intraperitoneal injection of lactated Ringer’s solution was given immediately after burn injury for fluid resuscitation. Animals were provided morphinated water ab libitum and monitored twice a day.

### Bacterial strains and growth conditions

*S*. *aureus* strains HG003 and JE-2 were cultured aerobically in Mueller-Hinton broth (MHB) at 37°C with shaking at 225 rpm. HG003 is a well-characterized model strain of *S*. *aureus*, while JE-2 is a well-characterized USA300 *S*. *aureus* associated with community-acquired MRSA infection. For anaerobic growth, overnight cultures were washed twice with PBS and diluted into 5 ml of pre-warmed (37°C) TSB + 100 mM MOPS (pH7) to an OD_600_ of 0.05. Cultures were prepared in triplicate in 16 x 150 mm glass tubes containing 1 mm stir bars. Following dilution, cultures were immediately transferred into a Coy anaerobic chamber and grown at 37°C with stirring. *P*. *aeruginosa* strains were grown aerobically in MHB at 37°C with shaking at 225 rpm. Burn wound isolates represent the first positive Pseudomonal wound cultures obtained from 5 unique patients admitted to the NC Jaycee Burn Center between Nov 2015 and April 2016 with a total body surface area burn ≥ 20% and/or inhalational injury after obtaining informed consent. CF isolates were collected from 5 patients at the UNC medical center. Isolates were cultured from sputum or bronchoalveolar lavage (BAL) from patients with CF after obtaining informed consent. All burn and CF isolates were grown on *Pseudomonas* isolation agar (BD Difco) and verified with 16S ribosomal sequencing using the primer pair 5’-AGTATTGAACTGAAGAGTTTGATCATGG-3’ and 5’-CTGAGATCTTCGATTAAGGAGGTGA-3’ for PCR amplification.

### Strain construction

The PAO1 *lasA* mutant (PW4282 *lasA*-H03∷ISlacZ/hah) was from the PAO1 knockout library [55]. PA14 deletion mutants were constructed by singly or sequentially deleting the coding sequences of *rhlA*, *pqsL*, *phzS*, and *hcnC*. Briefly, flanking primers were designed to anneal 800–1,200 bp upstream and downstream of the coding region. The resulting PCR product was inserted into plasmid pEX18Gm in accordance with the NEBuilder HiFi DNA Assembly protocol (New England Biolabs). Mutant alleles were integrated onto the chromosome of PA14 as described previously [56]. Briefly, pEX18Gm containing the in-frame deletion and gene-specific flanking regions was mated into *P*. *aeruginosa* via *Escherichia coli* S17-λpir. Primary integrants were selected for with gentamycin and irgasan, then grown for 4 h in LB without selection to allow for recombination. Dilutions of *P*. *aeruginosa* were plated on LB containing 8% sucrose for counterselection (loss of plasmid). Deletion strains were confirmed through PCR and sequencing (Genewiz). Plasmid P*pflB*∷*gfp* was constructed as follows, 298 bp upstream of the *pflB* coding region was amplified from HG003 genomic DNA using primers flanked with *EcoRI* and *XbaI* sites and cloned upstream of *gfpuvr* in plasmid pALC1434 [57].

### Antibiotic survival assays

To prepare sterile supernatants, *S*. *aureus* and *P*. *aeruginosa* strains were grown in MHB at 37°C with shaking at 225 rpm for approximately 20 h. The cultures were pelleted and supernatants were passed through a 0.2 μm filter. HG003 or JE-2 was grown to approximately 5 x 10^7^ (for cell wall acting antibiotics) or approximately 2 x 10^8^ cfu/ml (for all other antibiotics) in 3 ml MHB under aerobic or in 5 ml TSB + 100 mM MOPS under anaerobic conditions. Cells were pre-treated with 0.5 ml sterile supernatant (or 0.83 ml for anaerobic cultures) and returned to the incubator for a further 30 min. A 30-min pre-exposure was routinely used as we attempted to emulate the situation in vivo, where a population that has encountered and reacted to the relevant metabolites is subsequently exposed to antibiotic treatment. Where appropriate, cells were treated with 0.5 ml of *P*. *aeruginosa* culture taken directly from *s*tationary phase (18 h) cultures in place of sterile supernatant. Coculture experiments were performed in the presence of 5% bovine serum albumin (BSA) to facilitate *S*. *aureus/P*. *aeruginosa* coexistence. An aliquot was plated to enumerate cfu before the addition of antibiotics. Antibiotics were added at concentrations similar to the Cmax in humans at recommended dosing; ciprofloxacin 2.34 μg/ml [58], tobramycin 58 μg/ml [59], vancomycin 50 μg/ml [60]. The Cmax of vancomycin is physiologically relevant for bacteremia and infections with good blood supply. The Cmax of vancomycin in serum is likely higher than that reached in the lung during IV infusion, however, it is certainly within the range experienced during inhaled therapy where clinical trials observed a Cmax of 270 μg/ml in sputum of CF patients. The Cmax of tobramycin is 58 μg/ml. Regarding lung concentrations, work by Ruddy et al. has found that inhaled tobramycin therapy results in sputum concentrations of between 17.2 and 327.3 μg/ml. The concentration we use is well within this range. Also, this therapy fails to eradicate *P*. *aeruginosa* in vivo and thus, we believe, is physiologically relevant for this study [61]. The ciprofloxacin blood Cmax used in this study is 2.34 μg/ml. This is also well within the physiologically relevant concentration for lung infection where ciprofloxacin concentration is actually higher than corresponding blood serum levels [62].

Ciprofloxacin concentration was increased to 4.68 μg/ml when cells were grown in TSB + 100 mM MOPS to account for any decrease in pH where ciprofloxacin killing activity is reduced. At indicated times, an aliquot was removed and washed with 1% NaCl. Cells were serially diluted and plated to enumerate survivors. We routinely used 2 time points to enumerate survivors, 16 h and 24 h after antibiotic challenge as we previously found that, in *S*. *aureus*, susceptible cells are killed and a stable subpopulation of survivors emerges between 16 and 24 h of exposure to antibiotics [35,63]. Where indicated sterile supernatant was heat-inactivated at 95°C for 10 min before addition to culture. Where indicated, pyocyanin 100 μM, HQNO 11.5 μM, sodium cyanide 150 μM, or rhamnolipids 10–50 μg/ml (50/50 mix of mono- and di-rhamnolipids, Sigma) or L-rhamnose 10–50 μg/ml were added in place of supernatant. Concentrations of respiratory toxins represent levels detected in the sputum of CF patients [26,27].

### Promoter induction measurement

*S*. *aureus* strain HG003 harboring *gfp* promoter plasmid P*pflB*∷*gfp* was grown to approximately 2 x 10^8^ cfu/ml in 3 ml MHB containing chloramphenicol 10 μg/ml. Cultures were treated with 0.5 ml supernatant from HG003, PAO1, PA14, or *P*. *aeruginosa* clinical isolates as indicated. 200 μl culture was added to the wells of a clear bottom, black side 96-well plate. The plate was placed in a Biotek Synergy H1 microplate reader at 37°C with shaking. Absorbance (OD_600_) and GFP fluorescence (emission 528 nm and excitation 485 nm) were measured every 1 h for 16 h. GFP values were divided by OD_600_.

### ATP assays

HG003 was grown to approximately 2 x 10^8^ cfu/ml in 3 ml MHB and pre-treated with 0.5 ml sterile supernatant from *S*. *aureus* HG003 or *P*. *aeruginosa* PAO1 or PA14. ATP levels of the cultures were measured after 1.5 h, as described previously using a Promega BacTiter Glo kit according to the manufacturer’s instructions [35]. *P*-values are indicated.

### Vancomycin lysis assay

HG003 was grown to approximately 2 x 10^8^ cfu/ml in 3 ml MHB and pre-treated with 0.5 ml sterile supernatant from *S*. *aureus* HG003, *P*. *aeruginosa* PAO1, PA14, or *P*. *aeruginosa* clinical isolates as indicated. Cells were incubated for a further 30 min before addition of vancomycin 50 μg/ml. 200 μl aliquots were added to the wells of a clear 96-well plate and placed in a Biotek Synergy H1 microplate reader. Absorbance (OD_600_) was measured every 1 h for 16 h.

### Western blot analysis of LasA

*P*. *aeruginosa* strains were grown in MHB media for ~ 20 h. Cultures were normalized to OD_600_ 2.0 and pelleted, and supernatants were passed through a 0.2 μm filter. Supernatants were boiled in SDS-sample buffer and run on a 4%–12% bis-tris acrylamide gel (Invitrogen). Protein was transferred onto a PVDF membrane, and LasA was detected using rabbit polyclonal anti-LasA antibodies (LifeSpan BioSciences, Inc.).

### Staphylolytic assay

Staphylolytic assay was modified from Grande et al. [39]. Briefly, stationary phase *S*. *aureus* strain HG003 was heat killed at 95°C for 20 min. Cells were pelleted and resuspended in 20 mM Tris-HCl (pH 8.0) at an OD_595_ 0.8–1. *P*. *aeruginosa* strains were cultured in MHB media for approximately 20 h. Cultures were normalized to OD_600_ 2.0, pelleted and supernatants were passed through a 0.2 μm filter. Seventeen microliters of sterile supernatant were added to a 100 μl heat-killed cells. OD_595_ was measured at time 0 and after 2 h, and percent cell lysis was determined. The values shown represent the average of biological triplicates.

### Tobramycin-Texas Red uptake

Tobramycin-Texas Red was made as described previously [64,65]. *S*. *aureus* strain HG003 was grown to mid-exponential phase and then incubated with or without 30 μg/ml rhamnolipids for 30 min. Cells were plated to enumerate cfu prior to addition of Texas-Red tobramycin at a final concentration of 58 μg/ml. After 1 h, an aliquot of cells was removed, washed twice in 1% NaCl, and plated to enumerate survivors. The remaining aliquot was analyzed for Texas Red uptake on a BD Fortessa flow cytometer. Thirty thousand events were recorded. Figures were generated using FSC Express 6 Flow.

### Rhamnolipid quantification

*P*. *aeruginosa* rhamnolipid production was quantified utilizing a drop collapse assay, as previously described [33]. Briefly, clarified supernatants from overnight cultures of *P*. *aeruginosa* strains were serially diluted (1:1) with deionized water plus 0.005% crystal violet for visualization. Twenty-five microliters of aliquots of each dilution were spotted on to the underside of a petri dish plate and tilted to a 90° angle. Surfactant scores represent the reciprocal of the highest dilution at which a collapsed drop migrated down the surface of the plate.

### LC-MS/MS quantification of HQNO

Five hundred microliters of aliquots of *P*. *aeruginosa* supernatant were extracted 3 times with 1 ml of ethyl acetate containing 0.01% acetic acid. For each extraction, samples were vortexed for 30 s then centrifuged at 15,000 xg for 2 min. The organic phases were removed and combined in a separate tube and evaporated to dryness in a TurboVap under a gentle stream of nitrogen at 50°C. Dried samples were reconstituted in 250 μl acetonitrile and a portion diluted by a factor of 100 prior to analysis by liquid chromatography tandem mass spectrometry (LC-MS/MS). Quantitative analyses were performed on a Quantum Ultra triple quadrupole mass spectrometer (Thermo Scientific, Waltham, MA) equipped with an Acquity ultra-performance liquid chromatography (UPLC) system (Waters Corp., Milford, MA). A sample injection volume of 10 μl was separated on a 2.1 x 100 mm, 1.7 μm, CSH Fluoro-Phenyl UPLC column (Waters Corp., Milford, MA) at a flow rate of 250 μl per minute and a column temperature of 40°C. Mobile phase solvents consisted of 0.1% acetic acid in deionized water (A) and methanol (B). Separation was achieved with a linear gradient from 30% to 95% B over 5 min with a total run time of 10 min. Column effluent was diverted to waste from 1–3 and 7–10 min, and HQNO eluted at a retention time of 5.8 min. The mass spectrometer was operated in positive ion electrospray mode (3,000 V; 250°C), and data were acquired by selected reaction monitoring (SRM) in centroid mode using a mass transition of 260.1 to 159.3 m/z and a collision energy of 26 eV.

### Burn wound model of *P*. *aeruginosa/S*. *aureus* coinfection

Animals were purchased from Taconic Farms and housed in specific pathogen-free conditions in the Animal Association of Laboratory Animal Care at the University of North Carolina’s Department of Laboratory Animal Medicine Facilities. All protocols were approved by the Institutional Animal Care and Use Committee at the University of North Carolina, and all experiments were performed in accordance with the National Institutes of Health. Wild type C57BL/6 mice were burned and infected as previously described [41]. Briefly, a 65 g copper rod was heated to 100°C and used to create a full-contact burn of approximately 20% of total body surface area through 4 applications of the rod to the anesthetized animal’s dorsal region. In preparing the inoculum, overnight cultures of bacterial strains were subcultured into fresh MHB and grown for 2.5 h to mid-exponential phase. An aliquot of each culture was centrifuged and washed with 1 ml of PBS + 1% protease peptone. Approximate bacterial density was calculated by absorbance (OD_600_), and cultures were diluted to obtain the desired concentration. Inoculum was verified through CFU enumeration. Mice were infected subcutaneously in the mid-dorsal region in unburned skin surrounded by the wound 2 4h after burn. Mice were administered vancomycin intraperitoneally at 110 mg/kg once daily for 2 d, then sacrificed 48 h post infection. At the time of sacrifice, 5 mm tissue biopsy of the bacterial infection site were aseptically removed, homogenized with 3.2 mm stainless steel beads and a Bullet Blender (Next Advance; Averill Park, NY), then bacterial burden was enumerated by plating serial dilutions of the homogenates on selective media. Mice that mostly cleared *P*. *aeruginosa* (<10^3^ CFU/g tissue) were discounted from analysis.

### qRT-PCR

Two hundred and fifty microliters of homogenized tissue were suspended in 1 ml of Trizol (ThermoFisher) and frozen at −80°C until extraction. RNA was extracted following manufacturer’s protocol. Extracted RNA was DNase treated with 10 units RQ-1 RNase free DNase (Promega) following manufacturer’s protocol. RNA was purified after DNase treatment using RNA Clean and Concentrator-25 (Zymo) and eluted in 30 μl of H2O, and quantified using a NanoDrop spectrophotometer. Five hundred nanograms of total RNA were used to generate cDNA using SuperScript II Reverse Transcriptase (ThermoFisher) and Random Primer 9 (NEB) following manufacturer’s protocol. Copy number of *lasA* and *gyrA* were quantified using iTaq Universal Sybr Green master mix (Bio-Rad) in 20 ul reaction volumes on a Roche LightCycler 96, using the following primer pairs: lasA_RT_5 5’-CCTGTTCCTCTACGGTCGCG-3’, lasA_RT_3 5’-GGTTGATGCTGTAGTAGCCG-3’, gyrA_5_RT 5’-GAAGCTGCTCTCCGAATACC-3’, gyrA_3_RT 5’-CAGTTCCTCACGGATCACCT-3’.

### MIC assays

MICs were determined using the microdilution method. Briefly, approximately 5 x 10^5^ cfu were incubated with varying concentrations of ciprofloxacin, tobramycin, or vancomycin in a total volume of 200 μl MHB in a 96-well plate. Where indicated, 34 μl MHB was replaced with sterile *P*. *aeruginosa* or *S*. *aureus* supernatant or purified HQNO or rhamnolipids at final concentrations of 11.5 μM and 30 μg/ml, respectively. MICs were determined following incubation at 37°C for 24 h.

### Statistical analysis

Statistical data analysis was performed using Prism GraphPad software (San Diego, CA) version 5.0 b. Differences in *S*. *aureus* intracellular ATP concentration or surviving *S*. *aureus* CFU following *P*. *aeruginosa* supernatant treatment and antibiotic challenge were compared using a one-way ANOVA with Tukey’s multiple comparisons post test or the Student *t* test where appropriate. Differences in tissue burden following *S*. *aureus* and *P*. *aeruginosa* coinfection were compared with a Mann-Whitney test. Finally, the statistical significance of each correlation analysis was determined with a two-tailed Pearson’s chi-squared test. Differences with a *p*-value ≤ 0.05 were considered significant.

## Supporting information

S1 Table MICs of *S*. *aureus* HG003. MIC, minimum inhibitory concentration.
(DOCX)

S2 Table Summary of *P*. *aeruginosa* isolate phenotypes and resulting effect on HG003 susceptibility to listed antibiotics. (DOCX)

S1 Data Excel spreadsheet containing underlying data for Figs 1–5 and S1–S8 Figs. (XLSX)

S1 Fig Rhamnolipids do not cause cell death in *S*. *aureus* in the absence of tobramycin. *S*. *aureus* strain HG003 was grown to mid-exponential phase in MHB media and pre-treated with (A,B) sterile supernatants from *P*. *aeruginosa* PA14 wild type or isogenic mutants, *S*. *aureus* HG003 or (D) L-rhamnose 10–50 μg/ml before addition of tobramycin at 58 μg/ml. Where indicated, PA14 supernatant was heat inactivated (PA14 HI) at 95°C for 10 min. (C,D) Cultures were treated with exogenous rhamnolipids or L-rhamnose (10–50 μg/ml) in the absence of antibiotic. At indicated times, an aliquot was washed and plated to enumerate survivors. All experiments were performed in biological triplicate. Underlying data can be found in S1 Data. Error bars represent mean ± sd. MHB, Mueller-Hinton broth.
(TIF)

S2 Fig *Pseudomonas*-produced toxins inhibit respiration in *S*. *aureus* and induce antibiotic tolerance. (A) *S*. *aureus* HG003 was grown to mid-exponential phase in TSB + 100 mM MOPS in an anaerobic chamber and pre-treated with sterile supernatants from HG003 or PA14 for 30 min before addition of ciprofloxacin. HG003 was grown aerobically to mid-exponential phase in MHB media and pre-treated with (B) sterile supernatants from *P*. *aeruginosa* strains PA14 wild-type or its isogenic mutants or (C-D) physiologically relevant concentrations of HQNO, PYO, or NaCN for 30 min prior to antibiotic challenge. At indicated times, an aliquot was washed and plated to enumerate survivors. All experiments were performed in biological triplicate. Underlying data can be found in S1 Data. Error bars represent mean ± sd. HQNO, 4-hydroxyquinoline *N*-oxide; MHB, Mueller-Hinton broth; MOPS, 3-(*N*-morpholino)propanesulfonic acid; NaCN, sodium cyanide; PYO, pyocyanin; TSB, tryptic soy broth.
(TIF)

S3 Fig *P*. *aeruginosa* supernatant inhibits *S*. *aureus* aerobic respiration. (A-C) *S*. *aureus* strain HG003 harboring plasmid *PpflB*∷*gfp* was grown to mid-exponential phase and treated with supernatant from *P*. *aeruginosa* clinical isolates or laboratory strains. OD_600_ and *gfp* expression levels were measured every 30 min for 16 h using a Biotek Synergy H1 microplate reader. All experiments were performed in biological triplicate. Underlying data can be found in S1 Data. Error bars represent mean ± sd.
(TIF)

S4 Fig *P*. *aeruginosa* endopeptidase LasA induces lysis in *S*. *aureus*. *S*. *aureus* HG003 was grown to mid-exponential phase and exposed to sterile supernatants indicated for 30 min prior to addition of vancomycin 50 μg/ml. (A) At indicated times, an aliquot was removed, washed, and plated to enumerate survivors. (B) At 24 h post antibiotic treatment, the turbidity of cultures treated with supernatant from HG003 (red), *P*. *aeruginosa* CF isolates (blue), or burn isolates (green) was measured by absorbance at OD_600_. *p < 0.05 by one-way ANOVA and Tukey’s multiple comparisons post test. All experiments were performed in biological triplicate. Underlying data can be found in S1 Data. Error bars represent mean ± sd. CF, cystic fibrosis.
(TIF)

S5 Fig *P*. *aeruginosa* LasA lyses heat-killed *S*. *aureus*. (A,B) Heat killed *S*. *aureus* cells were incubated with supernatant from *P*. *aeruginosa* isolates in a 96-well plate. Lysis of *S*. *aureus* was monitored by measuring OD_595_ every 5 min for 2 h. All experiments were performed in biological triplicate. Underlying data can be found in S1 Data. Error bars represent mean ± sd.
(TIF)

S6 Fig Exposure to *P*. *aeruginosa* supernatant alters MRSA antibiotic susceptibility. *S*. *aureus* strain JE-2 was grown to mid-exponential phase and exposed to sterile supernatants from *P*. *aeruginosa* for 30 mins prior to the addition of (A) tobramycin 58 μg/ml or (B) vancomycin 50 μg/ml. At indicated times, an aliquot was removed, washed and plated to enumerate survivors. All experiments were performed in biological triplicate. Underlying data can be found in S1 Data. Error bars represent mean ± sd. MRSA, methicillin-resistant *S*. *aureus*.
(TIF)

S7 Fig *P*. *aeruginosa* LasA potentiates vancomycin killing of *S*. *aureus* during *P*. *aeruginosa/S*. *aureus* coculture. *S*. *aureus* strain HG003 was grown to mid-exponential phase, exposed to 0.5 ml of stationary phase culture from *P*. *aeruginosa* strains PAO1 or PAO1 *lasA*∷*tet* and 5% BSA for 30 mins prior to addition of vancomycin (50 μg/ml). At indicated times, an aliquot was removed, washed and plated on selective media to enumerate (A) *S*. *aureus* and (B) *P*. *aeruginosa* cells. All experiments were performed in biological triplicate. Underlying data can be found in S1 Data. Error bars represent mean ± sd.
(TIF)

S8 Fig Burned mice maintain a high burden of *P*. *aeruginosa* PAO1 WT and PAO1 *lasA*∷*tet* during co-infection. Approximately 1 x 10^5^ CFU *S*. *aureus* strain HG003 was administered subcutaneously alone or in combination with approximately 1 x 10^3^ CFU *P*. *aeruginosa* PAO1 or PAO1 *lasA*∷*tn* 24 h after burn. Mice were left untreated or administered 110 mg/kg vancomycin subcutaneously once daily for 2 d. Mice were sacrificed 48 h post infection. (A) Tissue biopsies at the site of infection were harvested, homogenized and *P*. *aeruginosa* burdens were each enumerated. Data for each group are compiled from 2 independent experiments. Underlying data can be found in S1 Data. WT, wild-type.
(TIF)

S9 Fig Correlation analysis of *P*. *aeruginosa* exoproduct production and impact on *S*. *aureus* antibiotic susceptibility. (A) HQNO production (measured by mass spectrometry), and (B) lytic activity (measured by staphylolytic assay) of *P*. *aeruginosa* laboratory strains PAO1 and PA14 and 12 clinical isolates were correlated to the isolate’s impact on *S*. *aureus* susceptibility to (A) ciprofloxacin or (B) vancomycin. The correlation coefficient and *p*-value for each analysis is shown. Statistical significance was determined using a two-tailed Pearson’s chi-squared test. The figures presented summarize data depicted in Figs 1–4.
(TIF)

## Abbreviations

BAL: bronchoalveolar lavage
BSA: bovine serum albumin
CF: cystic fibrosis
HCN: hydrogen cyanide
HQNO: 4-hydroxyquinoline *N*-oxide
LC-MS/MS: liquid chromatography tandem mass spectrometry
MHB: Mueller-Hinton broth
MIC: minimum inhibitory concentration
MRSA: methicillin-resistant *S*. *aureus*
*pflb*: pyruvate acetyltransferase
PMF: proton-motive force
SCV: small colony variant
SRM: selected reaction monitoring
UPLC: ultra-performance liquid chromatography
WT: wild-type
