# Supplementary material for: Pseudomonas aeruginosa exoproducts determine antibiotic efficacy against Staphylococcus aureus
Source: PLoS Biol. 2017 Nov 27;15(11):e2003981. doi: 10.1371/journal.pbio.2003981 (PMC5720819; doi:10.1371/journal.pbio.2003981)
Supplement: S1 Table — MIC, minimum inhibitory concentration. (DOCX) [file pbio.2003981.s001.docx]

| Antibiotic | Control | + HG003 supernatant | PAO1/ PA14 supernatant | PA14 *ΔpqsLphzShcnC* | PA14  *ΔrhlA* | PAO1  *lasA::tet* |
| --- | --- | --- | --- | --- | --- | --- |
| MIC ciprofloxacin (µg/ml) | 0.312 | 0.312 | 0.312 | 0.156 | 0.156 | 0.156 |
| MIC tobramycin (µg/ml) | 0.78 | 0.78 | 3.125-6.25 | 0.39 | 6.25 | 3.125 |
| MIC vancomycin (µg/ml) | 1.25 | 1.25 | 1.25 | 1.25 | 1.25 | 1.25 |

| Antibiotic | Control | 11.5µM HQNO | 30µg/ml rhamnolipids |
| --- | --- | --- | --- |
| MIC ciprofloxacin  (µg/ml) | 0.312 | 0.156 | 0.312 |
| MIC tobramycin (µg/ml) | 0.78 | 6.25 | 0.0975 |
| MIC vancomycin  (µg/ml) | 1.25 | 1.25 | 1.25 |

**Table S1. Minimum inhibitory concentrations (MIC) of *S. aureus* HG003**
