# Supplementary material for: Pseudomonas aeruginosa exoproducts determine antibiotic efficacy against Staphylococcus aureus
Source: PLoS Biol. 2017 Nov 27;15(11):e2003981. doi: 10.1371/journal.pbio.2003981 (PMC5720819; doi:10.1371/journal.pbio.2003981)
Supplement: S2 Table — (DOCX) [file pbio.2003981.s002.docx]

| **TTable S2. Summary of *P. aeruginosa* isolate phenotypes and resulting impact**  **on HG003 susceptibility to listed antibiotics** | | | | | | |  |
| --- | --- | --- | --- | --- | --- | --- | --- |
|  |  |  |  |  | | |  |
| **Strain** | **RL** | **HQNO** | **LasA** | **Tobramycin** | **Ciprofloxacin** | **Vancomycin** |  |
| PAO1 | + | + | + | nc | 🡻 | 🡹 |  |
| PA14 | + | + | + | nc | 🡻 | 🡹 |  |
| BC236 | - | - | - | nc | nc | nc |  |
| BC238 | + | + | + | nc | 🡻 | 🡹 |  |
| BC239 | - | + | - | 🡻 | 🡻 | nc |  |
| BC237 | + | + | - | nc | 🡻 | nc |  |
| BC308 | - | - | - | nc | nc | nc |  |
| BC310 | + | - | + | 🡹 | nc | 🡹 |  |
| BC312 | + | + | + | 🡻 | 🡻 | nc |  |
| BC249 | + | + | + | nc | 🡻 | 🡹 |  |
| BC250 | + | + | + | nc | 🡻 | 🡹 |  |
| BC251 | - | - | - | nc | nc | 🡹 |  |
| BC252 | + | + | + | 🡻 | 🡻 | 🡹 |  |
| BC253 | - | + | + | nc | 🡻 | nc |  |
| *RL= Rhamnolipid production | | | | | | |  |
|  |  |  |  |  |  |  | |
|  |  |  |  |  |  |  | |
