# Supplementary figures and images for: Pseudomonas aeruginosa exoproducts determine antibiotic efficacy against Staphylococcus aureus
Source: PLoS Biol. 2017 Nov 27;15(11):e2003981. doi: 10.1371/journal.pbio.2003981 (PMC5720819; doi:10.1371/journal.pbio.2003981)

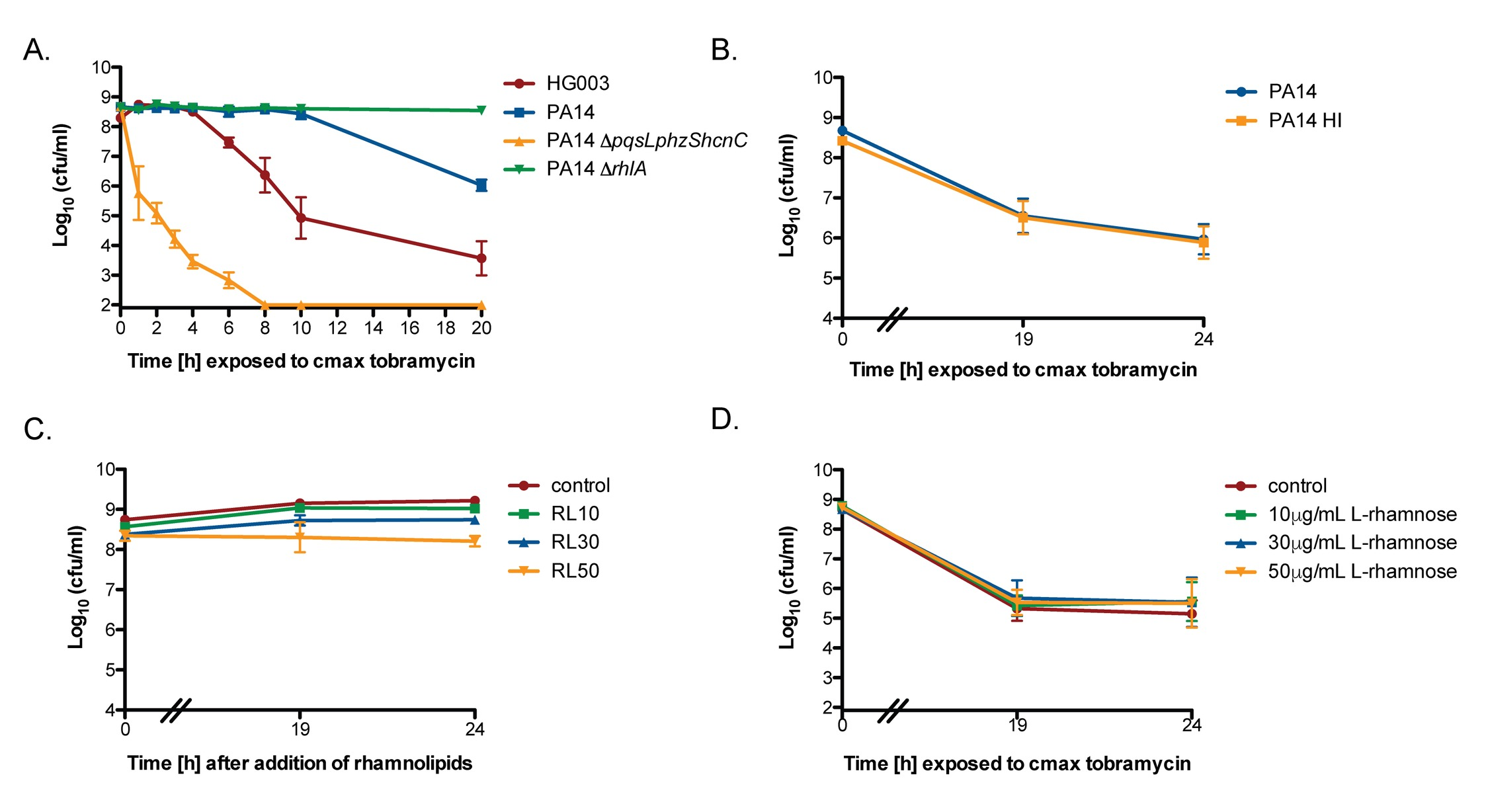

Supplement: S1 Fig — S. aureus strain HG003 was grown to mid-exponential phase in MHB media and pre-treated with (A,B) sterile supernatants from P. aeruginosa PA14 wild type or isogenic mutants, S. aureus HG003 or (D) L-rhamnose 10–50 μg/ml before addition of tobramycin at 58 μg/ml. Where indicated, PA14 supernatant was heat inactivated (PA14 HI) at 95°C for 10 min. (C,D) Cultures were treated with exogenous rhamnolipids or L-rhamnose (10–50 μg/ml) in the absence of antibiotic. At indicated times, an aliquot was washed and plated to enumerate survivors. All experiments were performed in biological triplicate. Underlying data can be found in S1 Data. Error bars represent mean ± sd. MHB, Mueller-Hinton broth. (TIF) [file pbio.2003981.s004.tif]

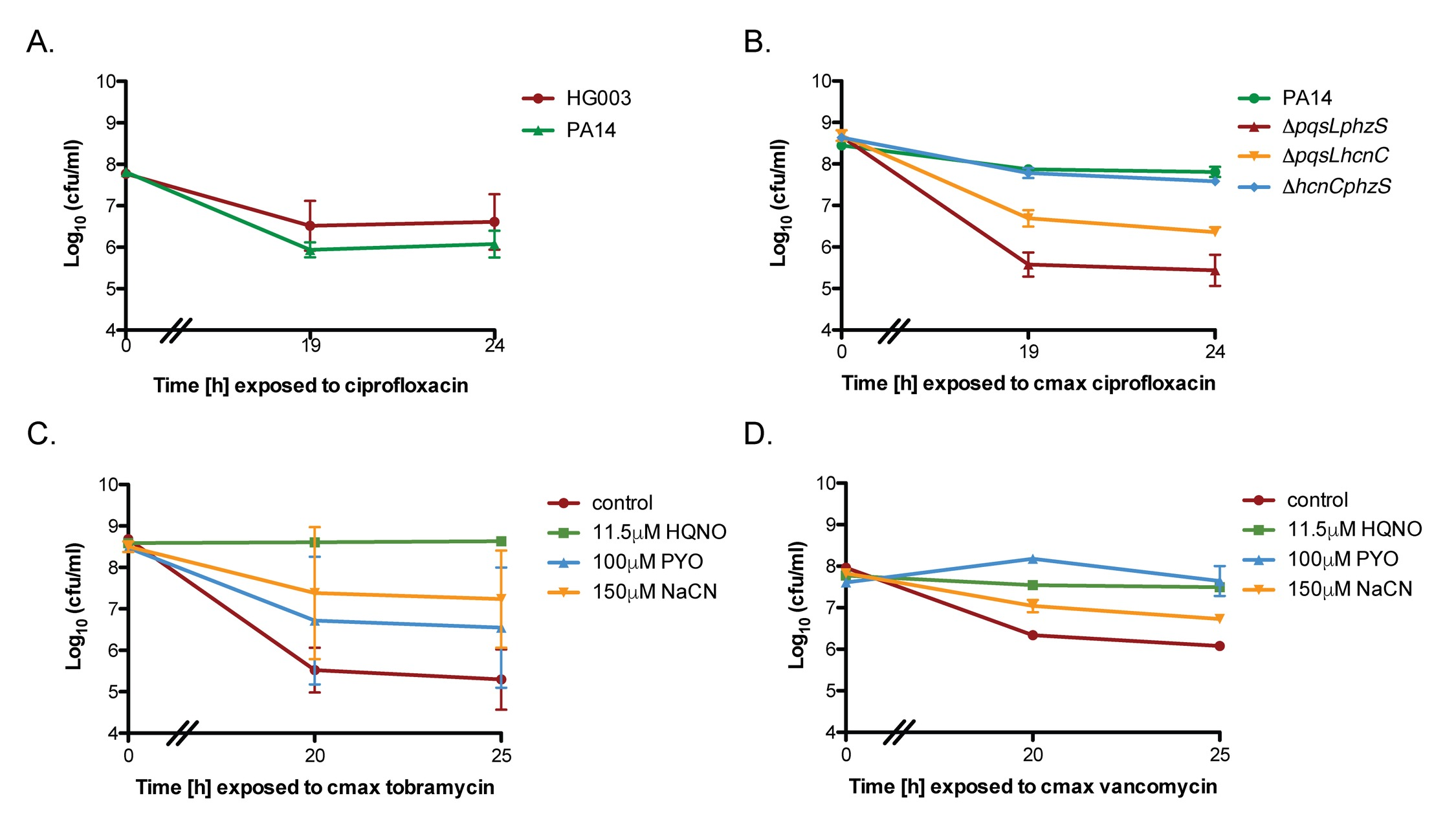

Supplement: S2 Fig — (A) S. aureus HG003 was grown to mid-exponential phase in TSB + 100 mM MOPS in an anaerobic chamber and pre-treated with sterile supernatants from HG003 or PA14 for 30 min before addition of ciprofloxacin. HG003 was grown aerobically to mid-exponential phase in MHB media and pre-treated with (B) sterile supernatants from P. aeruginosa strains PA14 wild-type or its isogenic mutants or (C-D) physiologically relevant concentrations of HQNO, PYO, or NaCN for 30 min prior to antibiotic challenge. At indicated times, an aliquot was washed and plated to enumerate survivors. All experiments were performed in biological triplicate. Underlying data can be found in S1 Data. Error bars represent mean ± sd. HQNO, 4-hydroxyquinoline N-oxide; MHB, Mueller-Hinton broth; MOPS, 3-(N-morpholino)propanesulfonic acid; NaCN, sodium cyanide; PYO, pyocyanin; TSB, tryptic soy broth. (TIF) [file pbio.2003981.s005.tif]

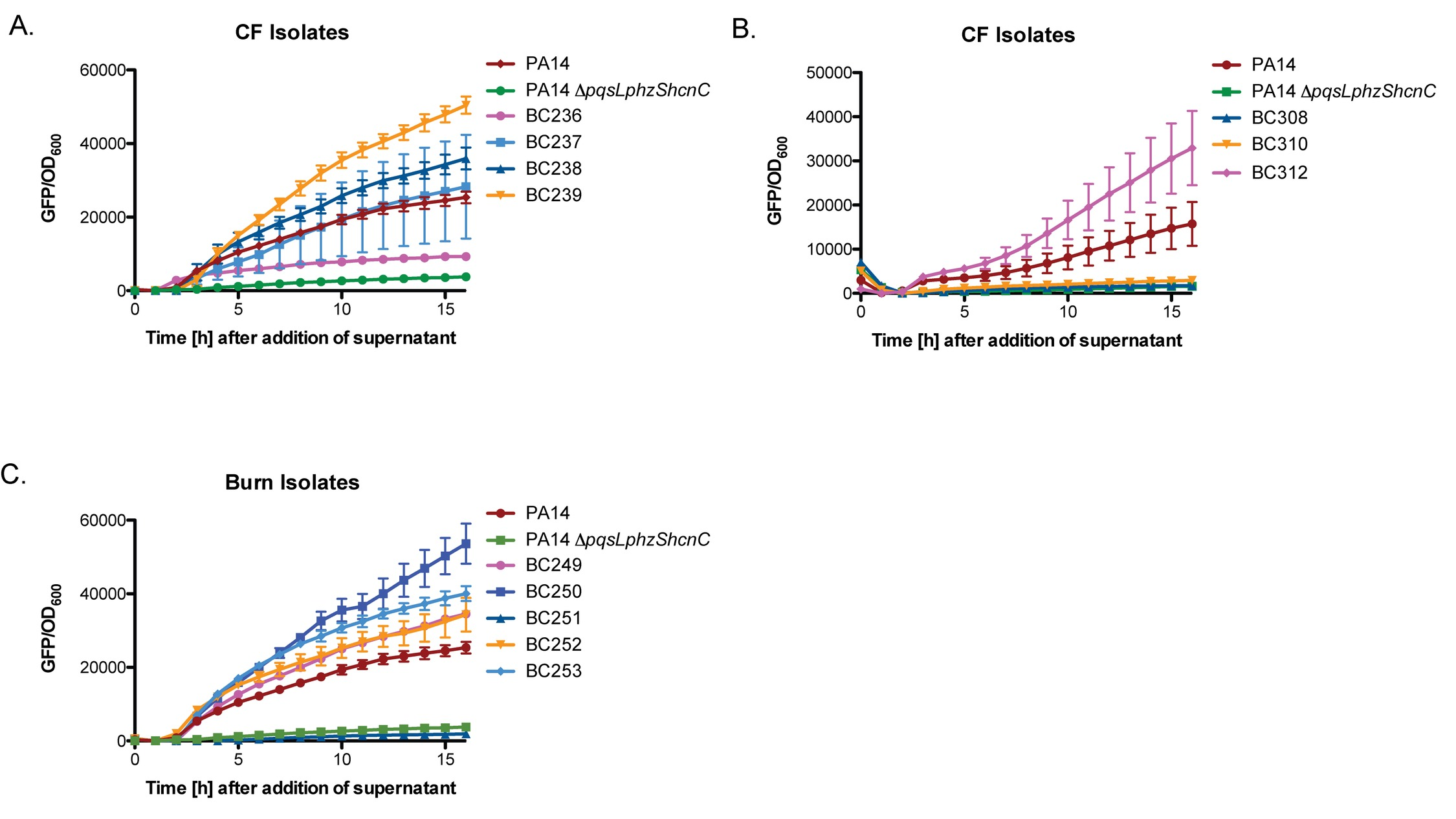

Supplement: S3 Fig — (A-C) S. aureus strain HG003 harboring plasmid PpflB∷gfp was grown to mid-exponential phase and treated with supernatant from P. aeruginosa clinical isolates or laboratory strains. OD600 and gfp expression levels were measured every 30 min for 16 h using a Biotek Synergy H1 microplate reader. All experiments were performed in biological triplicate. Underlying data can be found in S1 Data. Error bars represent mean ± sd. (TIF) [file pbio.2003981.s006.tif]

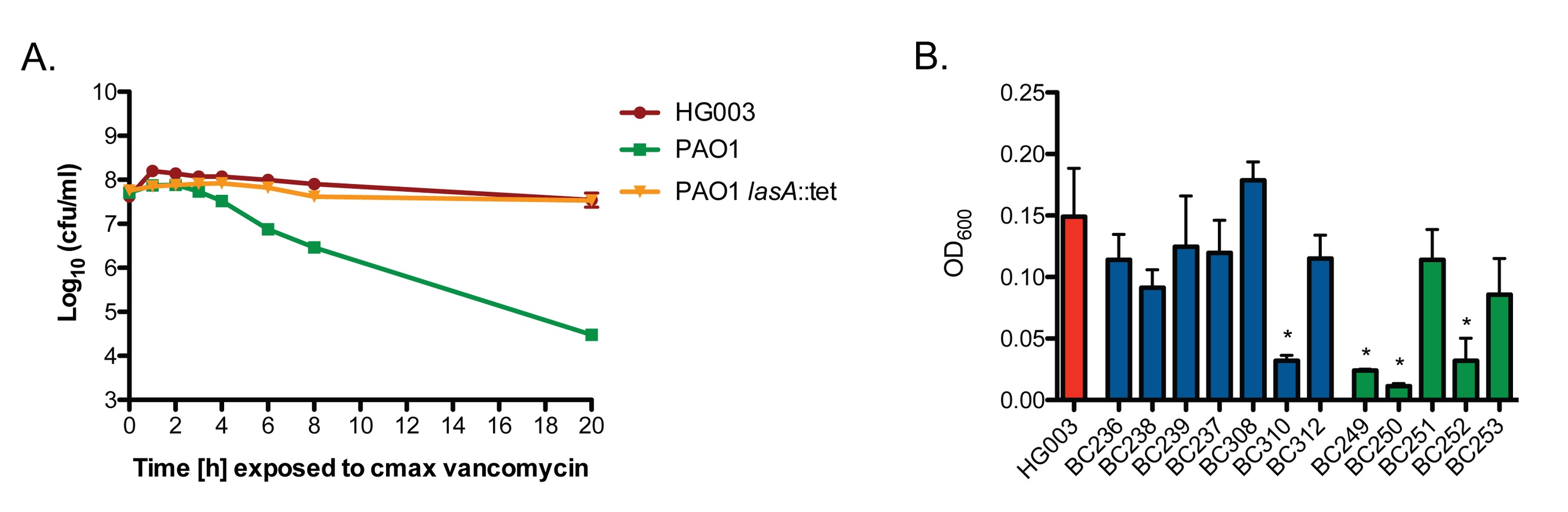

Supplement: S4 Fig — S. aureus HG003 was grown to mid-exponential phase and exposed to sterile supernatants indicated for 30 min prior to addition of vancomycin 50 μg/ml. (A) At indicated times, an aliquot was removed, washed, and plated to enumerate survivors. (B) At 24 h post antibiotic treatment, the turbidity of cultures treated with supernatant from HG003 (red), P. aeruginosa CF isolates (blue), or burn isolates (green) was measured by absorbance at OD600. *p < 0.05 by one-way ANOVA and Tukey’s multiple comparisons post test. All experiments were performed in biological triplicate. Underlying data can be found in S1 Data. Error bars represent mean ± sd. CF, cystic fibrosis. (TIF) [file pbio.2003981.s007.tif]

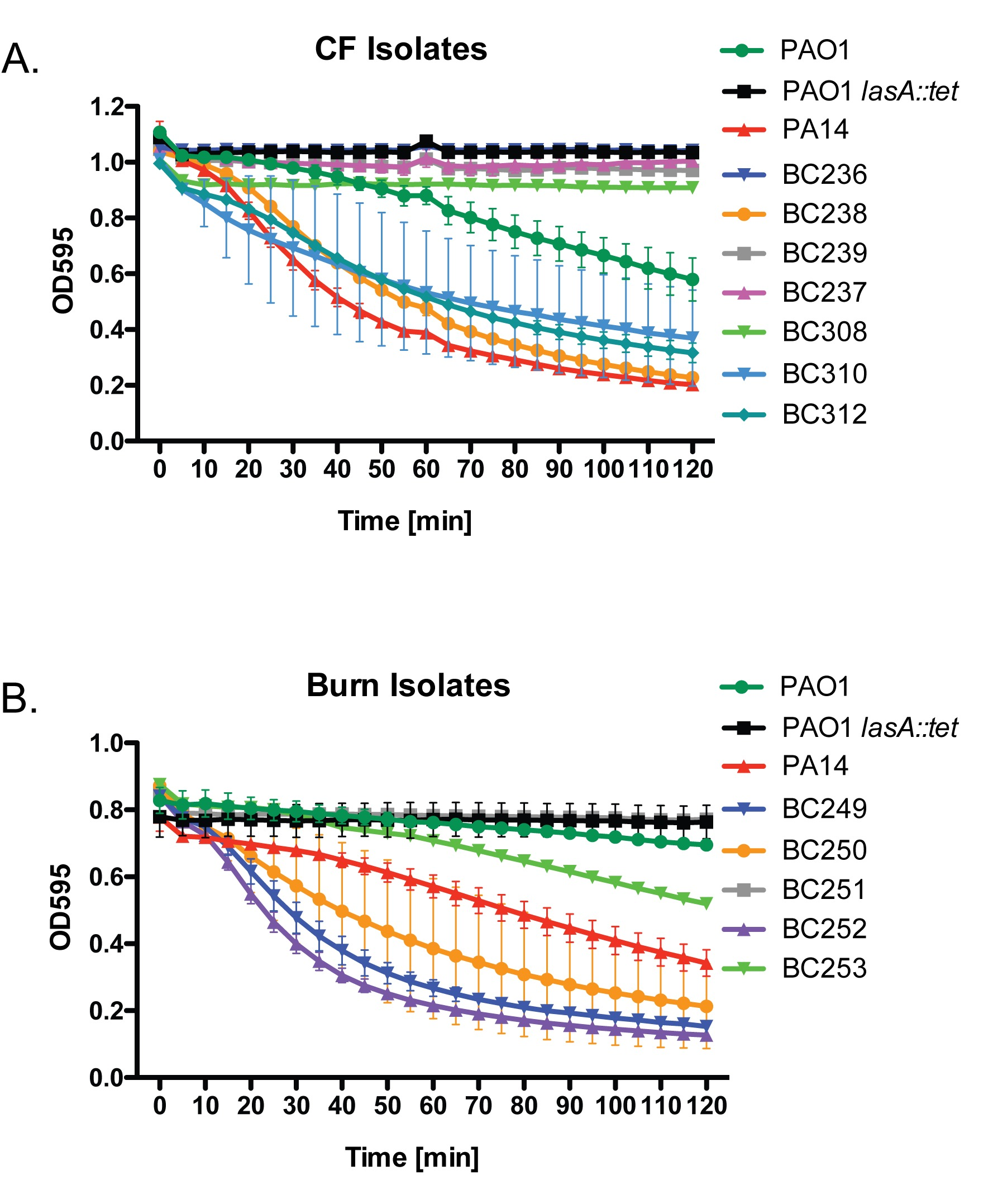

Supplement: S5 Fig — (A,B) Heat killed S. aureus cells were incubated with supernatant from P. aeruginosa isolates in a 96-well plate. Lysis of S. aureus was monitored by measuring OD595 every 5 min for 2 h. All experiments were performed in biological triplicate. Underlying data can be found in S1 Data. Error bars represent mean ± sd. (TIF) [file pbio.2003981.s008.tif]

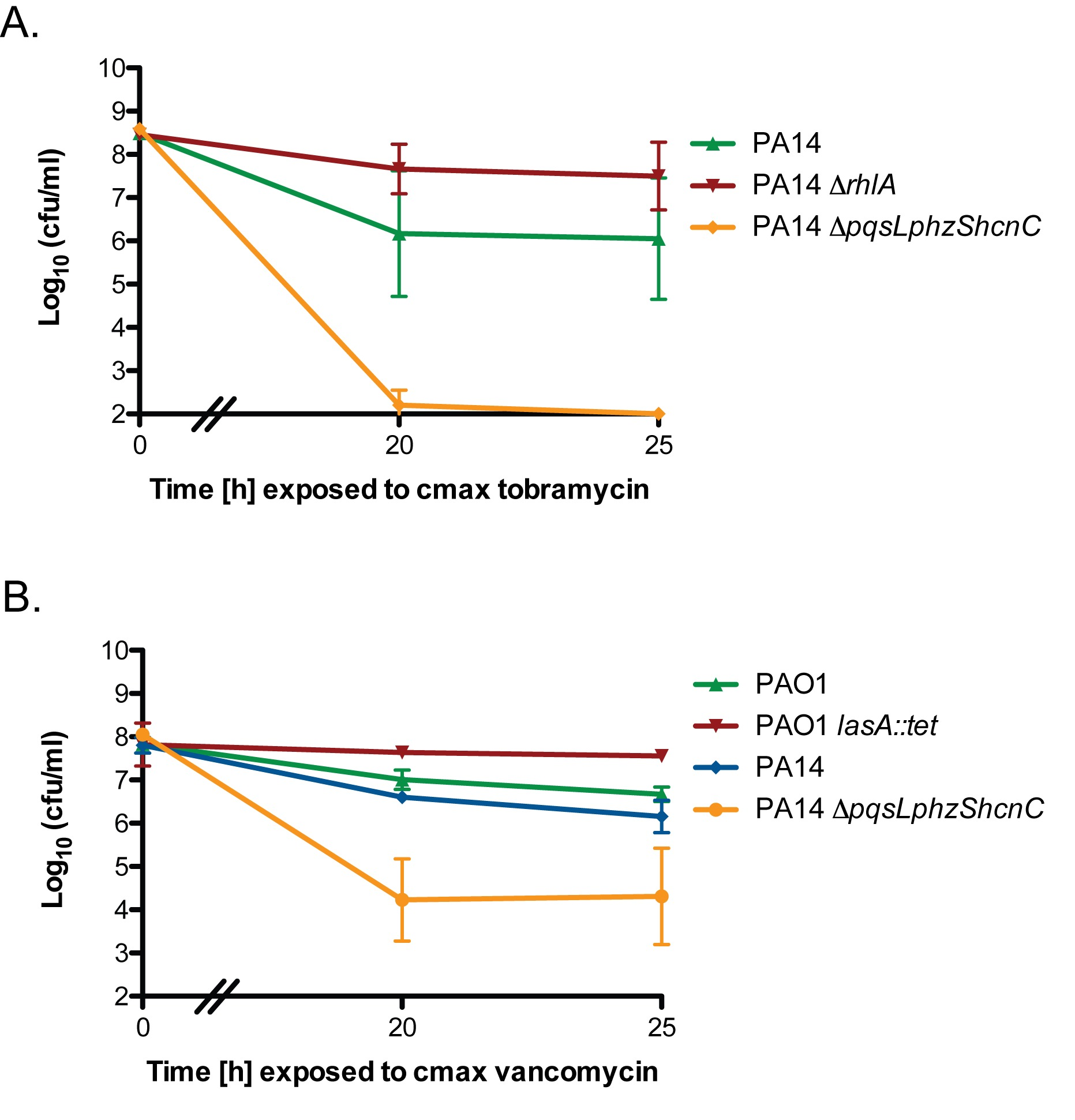

Supplement: S6 Fig — S. aureus strain JE-2 was grown to mid-exponential phase and exposed to sterile supernatants from P. aeruginosa for 30 mins prior to the addition of (A) tobramycin 58 μg/ml or (B) vancomycin 50 μg/ml. At indicated times, an aliquot was removed, washed and plated to enumerate survivors. All experiments were performed in biological triplicate. Underlying data can be found in S1 Data. Error bars represent mean ± sd. MRSA, methicillin-resistant S. aureus. (TIF) [file pbio.2003981.s009.tif]

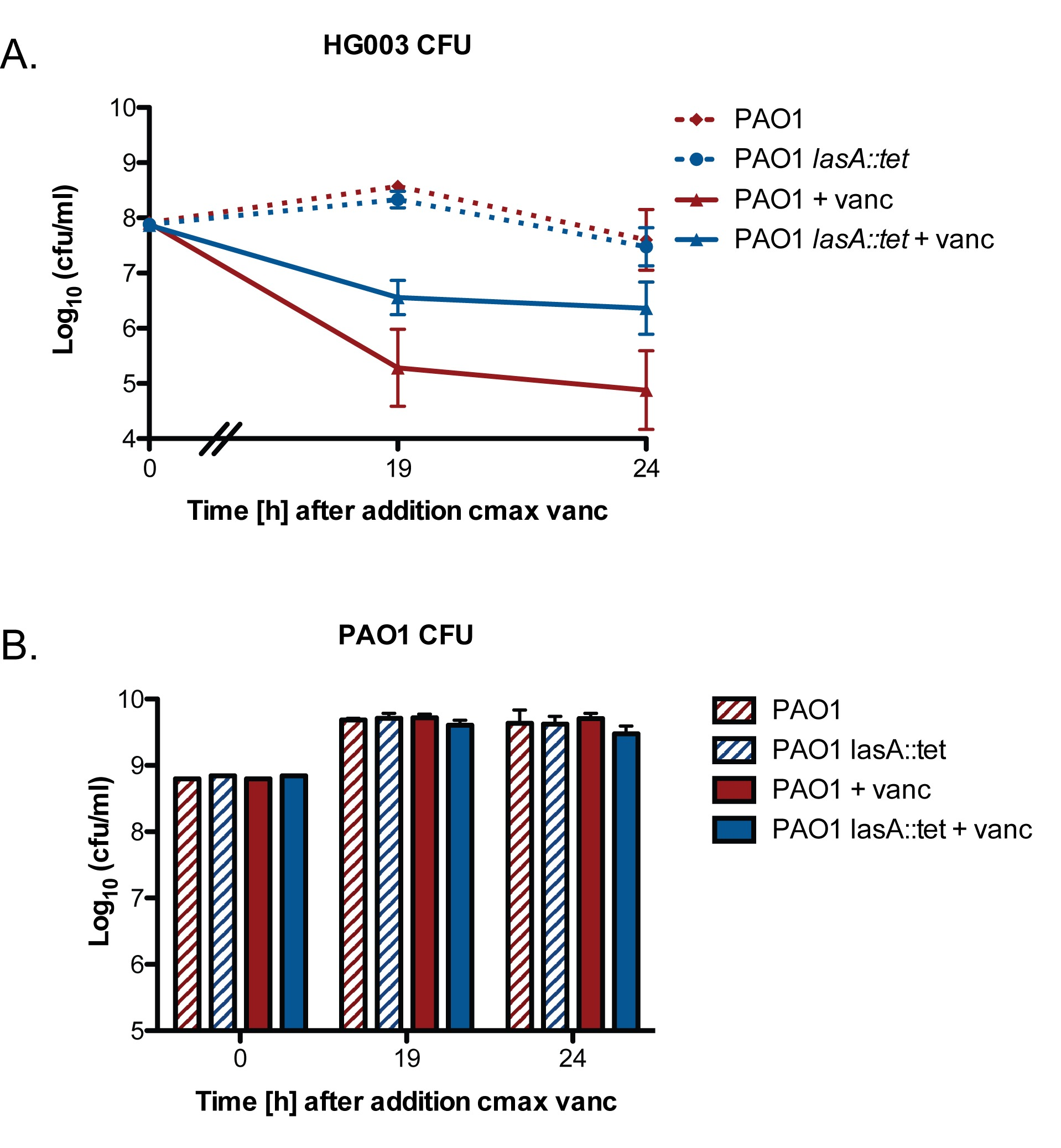

Supplement: S7 Fig — S. aureus strain HG003 was grown to mid-exponential phase, exposed to 0.5 ml of stationary phase culture from P. aeruginosa strains PAO1 or PAO1 lasA∷tet and 5% BSA for 30 mins prior to addition of vancomycin (50 μg/ml). At indicated times, an aliquot was removed, washed and plated on selective media to enumerate (A) S. aureus and (B) P. aeruginosa cells. All experiments were performed in biological triplicate. Underlying data can be found in S1 Data. Error bars represent mean ± sd. (TIF) [file pbio.2003981.s010.tif]

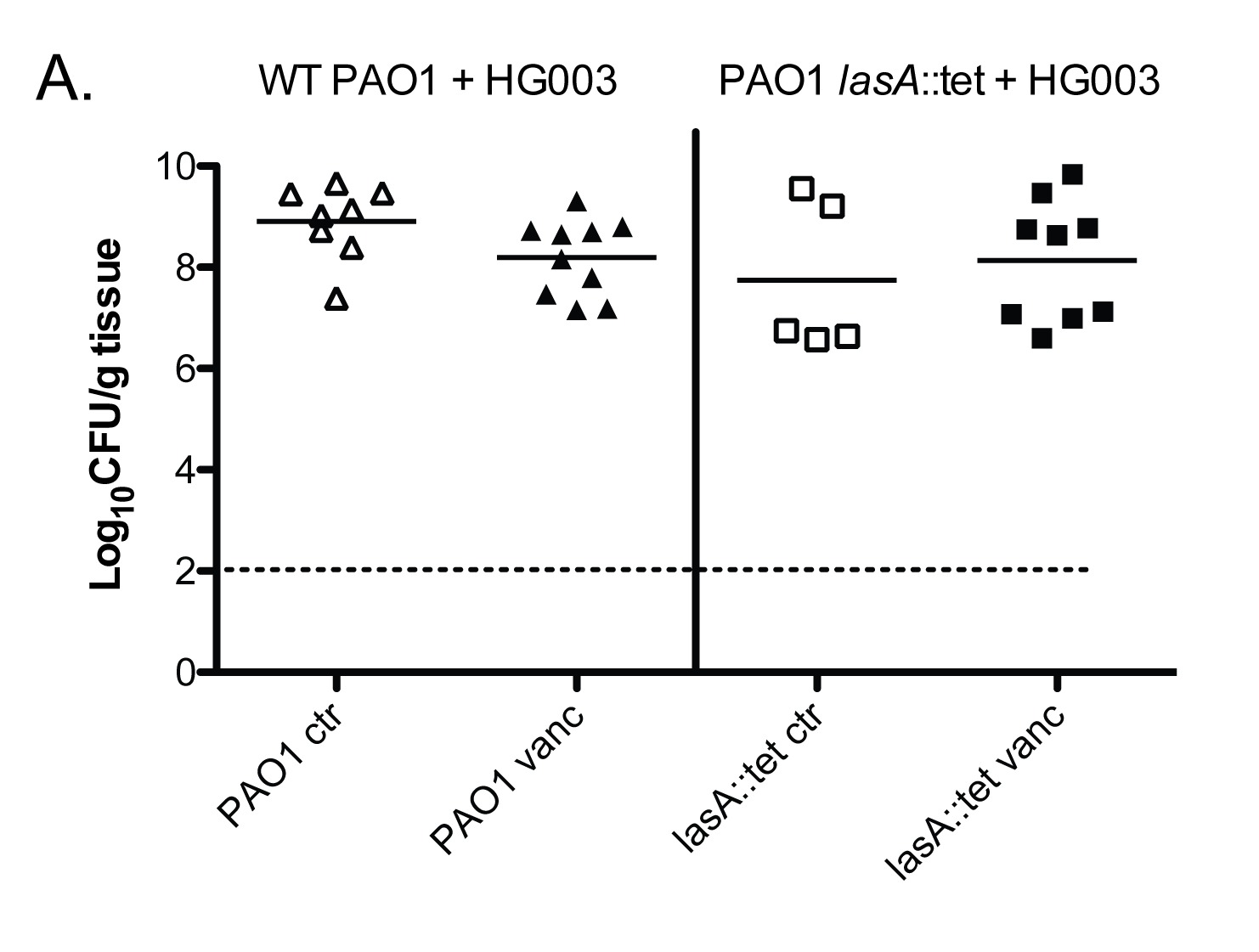

Supplement: S8 Fig — Approximately 1 x 10^5^ CFU S. aureus strain HG003 was administered subcutaneously alone or in combination with approximately 1 x 10^3^ CFU P. aeruginosa PAO1 or PAO1 lasA∷tn 24 h after burn. Mice were left untreated or administered 110 mg/kg vancomycin subcutaneously once daily for 2 d. Mice were sacrificed 48 h post infection. (A) Tissue biopsies at the site of infection were harvested, homogenized and P. aeruginosa burdens were each enumerated. Data for each group are compiled from 2 independent experiments. Underlying data can be found in S1 Data. WT, wild-type. (TIF) [file pbio.2003981.s011.tif]

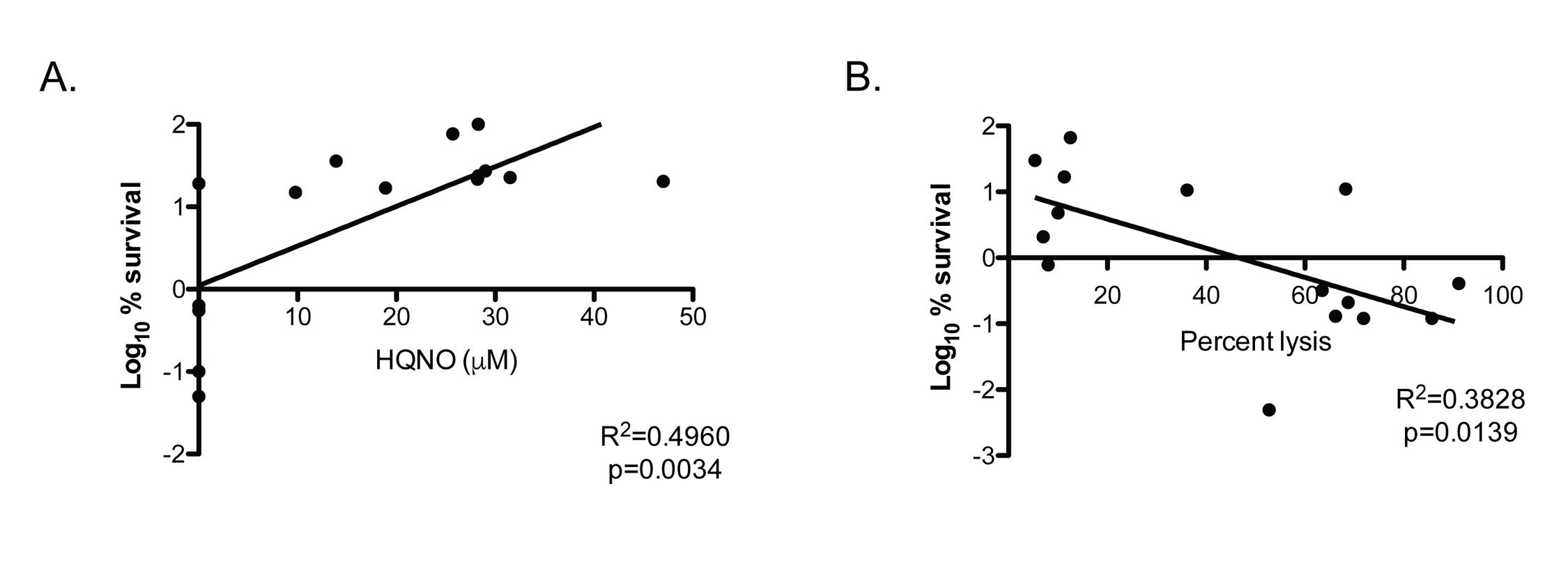

Supplement: S9 Fig — (A) HQNO production (measured by mass spectrometry), and (B) lytic activity (measured by staphylolytic assay) of P. aeruginosa laboratory strains PAO1 and PA14 and 12 clinical isolates were correlated to the isolate’s impact on S. aureus susceptibility to (A) ciprofloxacin or (B) vancomycin. The correlation coefficient and p-value for each analysis is shown. Statistical significance was determined using a two-tailed Pearson’s chi-squared test. The figures presented summarize data depicted in Figs 1–4. (TIF) [file pbio.2003981.s012.tif]
